# Identification of Essential Genes in the Salmonella Phage SPN3US Reveals Novel Insights into Giant Phage Head Structure and Assembly

**DOI:** 10.1128/JVI.01492-16

**Published:** 2016-10-28

**Authors:** Julie A. Thomas, Andrea Denisse Benítez Quintana, Martine A. Bosch, Adriana Coll De Peña, Elizabeth Aguilera, Assitan Coulibaly, Weimin Wu, Michael V. Osier, André O. Hudson, Susan T. Weintraub, Lindsay W. Black

**Affiliations:** a Thomas H. Gosnell School of Life Sciences, Rochester Institute of Technology, Rochester, New York, USA; b Natural and Physical Sciences, Baltimore City Community College, Baltimore, Maryland, USA; c National Institute of Arthritis and Musculoskeletal and Skin Diseases, National Institutes of Health, Bethesda, Maryland, USA; d University of Texas Health Science Center at San Antonio, San Antonio, Texas, USA; e University of Maryland School of Medicine, Baltimore, Maryland, USA; University of California, Irvine

## Abstract

Giant tailed bacterial viruses, or phages, such as Pseudomonas aeruginosa phage ϕKZ, have long genomes packaged into large, atypical virions. Many aspects of ϕKZ and related phage biology are poorly understood, mostly due to the fact that the functions of the majority of their proteins are unknown. We hypothesized that the Salmonella enterica phage SPN3US could be a useful model phage to address this gap in knowledge. The 240-kb SPN3US genome shares a core set of 91 genes with ϕKZ and related phages, ∼61 of which are virion genes, consistent with the expectation that virion complexity is an ancient, conserved feature. Nucleotide sequencing of 18 mutants enabled assignment of 13 genes as essential, information which could not have been determined by sequence-based searches for 11 genes. Proteome analyses of two SPN3US virion protein mutants with knockouts in *64* and *241* provided new insight into the composition and assembly of giant phage heads. The *64* mutant analyses revealed all the genetic determinants required for assembly of the SPN3US head and a likely head-tail joining role for gp64, and its homologs in related phages, due to the tailless-particle phenotype produced. Analyses of the mutation in *241*, which encodes an RNA polymerase β subunit, revealed that without this subunit, no other subunits are assembled into the head, and enabled identification of a “missing” β′ subunit domain. These findings support SPN3US as an excellent model for giant phage research, laying the groundwork for future analyses of their highly unusual virions, host interactions, and evolution.

**IMPORTANCE** In recent years, there has been a paradigm shift in virology with the realization that extremely large viruses infecting prokaryotes (giant phages) can be found in many environments. A group of phages related to the prototype giant phage ϕKZ are of great interest due to their virions being among the most complex of prokaryotic viruses and their potential for biocontrol and phage therapy applications. Our understanding of the biology of these phages is limited, as a large proportion of their proteins have not been characterized and/or have been deemed putative without any experimental verification. In this study, we analyzed Salmonella phage SPN3US using a combination of genomics, genetics, and proteomics and in doing so revealed new information regarding giant phage head structure and assembly and virion RNA polymerase composition. Our findings demonstrate the suitability of SPN3US as a model phage for the growing group of phages related to ϕKZ.

## INTRODUCTION

“Giant” and “jumbo” bacterial viruses are terms used to describe tailed phages with long double-stranded DNA (dsDNA) genomes (>200 kb) and large, structurally complex virions (1, 2). For many years, giant phages were considered elaborate oddities of little general relevance due to their perceived rarity (1). However, this viewpoint has changed radically in the last decade with the realization that standard phage isolation techniques were biased against larger phages and that giant phages can be readily isolated from a diversity of environmental samples (3–5). The first giant phage genome sequenced was the 280-kb genome of Pseudomonas aeruginosa ϕKZ (2). There has been much interest in ϕKZ and related phages for their clinical use in the treatment of multidrug-resistant bacteria, i.e., phage therapy (6–9). The virion of ϕKZ is of great interest, as it is one of the most complex of known prokaryotic viruses, having a large T=27 capsid (10, 11) and a complex baseplate (12). Notably, in the ϕKZ virion, there is an unusual structural feature, known as the inner body (IB), that is not seen in other phage types. The IB forms a large cylindrical proteinaceous structure within the head around which the dsDNA is spooled (13). The three-dimensional reconstruction of the ϕKZ IB enabled estimation that it is composed of a 15- to 20-MDa protein, all of which is likely ejected into the host cell with the genome (14). This indicates that the IB likely has a novel role(s) in host takeover, as well as virion assembly and stability (15, 16). Mass spectrometry (MS) of the ϕKZ head identified candidates for the IB proteins, none of which can be assigned a function using bioinformatics (15).

Another unusual feature of ϕKZ and related phages is the presence of two multisubunit RNA polymerases (RNAPs)—quite different from the single-subunit RNAPs encoded by phages T7 and N4. The existence of enough β and β′ subunits to account for two separate RNAPs was first demonstrated for Pseudomonas chlororaphis phage 201ϕ2-1 (which belongs to the ϕKZ-like phage genus) using bioinformatics and mass spectrometry analyses (17). One of the RNAPs of these giant phages, the virion RNAP (vRNAP), is packaged into the phage head and is ejected into the host cell with the phage DNA for the transcription of early phage genes (17). The second RNAP, the nonvirion RNAP (nvRNAP), was recently purified from cells infected with ϕKZ and shown to be responsible for the transcription of late genes (18). There is great interest in the unusual RNAPs of these phages, as ϕKZ is able to infect its host in the presence of rifampin, indicating that phage transcription could be completely independent of the host transcriptional machinery (19).

The first phages related to ϕKZ whose genomes were sequenced infected pseudomonads (9, 17, 20, 21), but recent studies have shown a broader host diversity of related phages, such as Salmonella phage SPN3US, Vibrio phage JM-2012, and Erwinia amylovora phage Ea35-70 (9, 17, 20, 22, 23). Recently, the giant Bacillus subtilis phage AR9, whose 251-kb genome includes 292 open reading frames (ORFs), was also described as ϕKZ related, as it shares a core set of orthologous genes with sequenced phages related to ϕKZ, including homologs of the vRNAP and nvRNAP (24). The isolation of increasing numbers of giant phages indicates that further evolutionary and taxonomic clarification of these phages is required. Importantly, the existence of these phages raises many questions pertaining to what makes a giant phage a “giant,” their host interactions (and how we can exploit them most effectively for phage therapy), their roles in the environment, and their evolution.

These questions are not straightforward to address, as the genomes of the giant phages related to ϕKZ range in length from approximately 167 kb (JM-2012) to 316 kb (201ϕ2-1) and include many genes that are highly divergent in nature compared to those of other phage groups (17, 25). This divergence makes functional annotation of proteins very difficult, even for proteins that are essential and well conserved in many tailed phage types and whose identification is normally straightforward—for instance, it was 10 years after ϕKZ was first sequenced that its portal protein and split subunit DNA polymerase were identified (22), as well as its prohead protease (15). Comparative genome analyses have revealed an unusually high degree of genome rearrangement, especially inversions, between different members of the ϕKZ-related phages (17, 22, 25). Obviously, with many genes whose functions remain undetermined, the ϕKZ-related phages are likely to encode many novel molecular mechanisms, and the challenge is how to unravel them.

One approach to understanding phage infections is to use omics approaches, including transcriptomics and metabolomics (26, 27); however, seeing a clear link between any individual phage gene and its functions is not always straightforward. Historically, the genetic system based on the T4 phage has been one of the most paradigm-shifting model systems in biology, and it is critical for our understanding of the fine structure of the gene and many of the founding principles of molecular genetics (28–31). The T4 system continues to be employed by researchers around the world for purposes ranging from addressing questions pertaining to fundamental molecular interactions (such as the molecular motor that drives DNA into phage heads [32]) to the creation of novel vaccines and therapeutics (33, 34). We hypothesized that a similar model system for a giant phage would also be an extremely powerful tool for understanding their biology and potentially for developing novel therapeutics, especially if we could blend classical genetics with omics approaches. The isolation of phage SPN3US by Lee et al. (35) created an opportunity for us to test our hypothesis, as SPN3US infects Salmonella, a genetic workhorse with its well-characterized genome and suppressor strains (36, 37). We were particularly interested in testing our hypothesis to identify a phage-host system in which we could study giant phage head assembly and structure.

In this study, we show that SPN3US contains a set of genes homologous to those of ϕKZ and other giant phages. We demonstrate that SPN3US amber mutants that have mutations in these conserved genes can be isolated, indicating that the analysis of such mutants will have broader relevance. The proteomes of two SPN3US head mutants have revealed novel findings pertaining to giant phage head assembly and structure and to transcription.

## MATERIALS AND METHODS

### Identification of homologs of SPN3US proteins in other giant phages.

Proteins with similarity to those encoded by SPN3US (GenBank accession no. JN641803) and other long-genome phages were identified using CoreGenes (38, 39) and PsiBlast searches (40). CoreGene version 3.5 matches were determined using software available online (http://binf.gmu.edu:8080/CoreGenes3.5/BatchCoreGenes.html) with the default BLASTP threshold score of 75. The phage genome GenBank accession numbers used for the CoreGenes analyses were as follows: PhiEaH2, JX316028; CR5, JX094500; ϕKZ, AF399011.1; 201ϕ2-1, EU197055.1; and ϕPA3, HQ630627.1. PSI-BLAST (40) matches to SPN3US proteins were generated using a locally implemented version of the software with the entire NCBI nonredundant (nr) and environmental protein (env_nr) databases.

### Bacteria and phages.

The wild-type SPN3US phage was provided by Sangryeol Ryu (Seoul National University). The Salmonella enterica serovar Typhimurium LT2 nonsuppressor (sup−) TT9079 [genotype: *sty*(LT2) *hisC527*(UAG) *leuA414*(UAG) *srl-202*::Tn*10 recA1*] and suppressor (*supD*) TT6675 [genotype: *sty*(LT2) *hisC527*(UAG) *leuA414*(UAG) *supD10*(UAG, ser) *srl-202*::Tn*10 recA1*] strains were kindly provided by John Roth (University of California Davis). These strains and others in the Roth strain collection can be searched online (http://rothlab.ucdavis.edu/textStrainer). S. enterica serovar Typhimurium LT2 strain UB0015 (sup−) was provided by Sherwood Casjens (University of Utah). The bacterial stocks and phage were propagated using LB medium. Phages were propagated in overlays containing 0.34% agar at 30°C. Phage dilutions were prepared in SM buffer (50 mM Tris-HCl, pH 7.5, 100 mM sodium chloride, 10 mM magnesium sulfate, 0.01% [w/v] gelatin).

### Isolation of SPN3US amber mutant phage candidates.

Hydroxylamine (HA) mutagenesis of a high-titer stock (∼1 × 10^12^ PFU/ml) of SPN3US was performed as described for the T4 phage (41). The phage sample was treated at 37°C in the presence of 0.05 M sodium phosphate buffer (pH 6.0), 0.4 M hydroxylamine, and 1 mM EDTA. A control was included in which HA was replaced with SM buffer. At 23 h, aliquots of HA-treated and control suspensions were diluted 100-fold in LB broth supplemented with 1 mM EDTA. HA-treated samples determined to have an approximately 1,000-fold reduction in titer were either enriched by growth in a suppressor host (*supD*) and plated to obtain 100 to 200 plaques per overlay or plated directly to obtain individual plaques. Individual plaques were stabbed and transferred to suppressor and nonsuppressor host overlays, and after overnight incubation, they were examined for amber mutant candidate phages (i.e., those able to propagate only on the permissive [sup+] host). These isolates were retested to ensure the desired plating characteristics held, and high-titer stocks were prepared from individual plaques.

### DNA extraction of SPN3US mutant candidates.

SPN3US mutant phage DNA was extracted from high-titer stocks (typically 10^11^ to 10^12^ PFU/ml). DNA extracted from the mixture of SPN3US mutants was purified using organic solvent extraction followed by ethanol precipitation (42). DNA used for sequencing of the individual and two double mixtures of amber mutant phages was purified using a phage DNA isolation kit (Norgen).

### Sequencing of SPN3US mutant candidates.

DNA sequencing of the mixture of 50 DNAs was undertaken at LC Sciences (Houston, TX) on an Illumina HiSeq. Analyses to identify amber mutations in this mixture were performed by Accura Science. Individual mutant samples and two mixtures comprising two mutants each were prepared using the NexteraXT workflow. Three individual genomes and the two mixtures were sequenced on an Illumina HiSeq-2500 machine. Eleven other individual mutant phage genomes were sequenced on an Illumina MiSeq (150-bp paired-end reads). These 14 individual mutants and two mixtures were sequenced at the University of Rochester Genomics Research Center. Assemblies and single nucleotide polymorphism (SNP) analyses were performed using SeqMan NGen and SeqMan Pro, respectively (DNAStar). The wild-type sequence (GenBank accession no. JN641803.1) was used as the reference genome.

### Purification of SPN3US mutants.

Liquid cultures of S. enterica serovar Typhimurium LT2 TT6675 and TT9079 were grown to an optical density at 600 nm (OD_600_) of 0.3 at 30°C, infected at a multiplicity of infection (MOI) of 10 with an amber mutant phage, and propagated overnight. Samples were treated with lysozyme (0.5 mg/ml) on ice for 1 h and then spun at 4,300 × *g* for 10 min. The supernatant was decanted and then spun at 39,000 × *g* for 30 min at 4°C. The pellets were resuspended in SM buffer overnight at 4°C, and samples were further purified by ultracentrifugation on CsCl step and buoyant-density gradients. Phage samples (typically 300 μl) were layered onto CsCl step gradients composed of the following concentrations of CsCl: 1.59 g/ml (1 ml), 1.52 g/ml (1 ml), 1.41 g/ml (0.9 ml), 1.30 g/ml (0.9 ml), and 1.21 g/ml (0.9 ml). The buffer used throughout the gradient was 10 mM Tris-HCl (pH 7.5) and 1 mM MgCl_2_. The tubes were spun at 31,000 rpm for 3 h at 6°C in an SW50.1 rotor (Beckman Coulter ultracentrifuge), and the resulting bands were harvested by side tube puncture. The refractive index of each sample was measured using a refractometer, and then the sample was added to a freshly prepared solution of 10 mM Tris-HCl (pH 7.5) and 1 mM MgCl_2_ containing CsCl at the refractive index of each sample. The buoyant-density gradients then underwent overnight centrifugation at 31,000 rpm at 4°C. Samples were again collected by side tube puncture, and the refractive index was recorded and then dialyzed against three changes of 50 mM Tris-Cl (pH 7.5), 200 mM NaCl, and 10 mM MgCl_2_.

### Mass spectrometry of SPN3US mutants.

Samples from SPN3US mutants grown on S. enterica serovar Typhimurium LT2 strains TT6675 and TT9079 that had undergone purification and dialysis were boiled for 10 min in SDS sample buffer (Bio-Rad). The samples then underwent electrophoresis on Criterion XT MOPS (morpholinepropanesulfonic acid) SDS-12% PAGE reducing gels (Bio-Rad) and subsequent protein visualization by staining with Coomassie blue. The gel lanes were divided into six slices (see Fig. 4). Efforts were made to avoid transecting visibly stained bands. After destaining, proteins in the gel slices were reduced with TCEP [tris(2-carboxyethyl)phosphine hydrochloride] and then alkylated with iodoacetamide before digestion with trypsin (Promega). The gel slices were destained in 40 mM NH_4_CO_3_-50% acetonitrile, dehydrated in acetonitrile, and digested overnight at 37°C with trypsin (Promega; sequencing grade) in 40 mM NH_4_CO_3_-10% acetonitrile. The tryptic peptides were extracted with 0.1% trifluoroacetic acid (TFA) followed by 0.1% TFA-50% acetonitrile. The combined extracts were dried by vacuum centrifugation and resuspended in 0.5% TFA for analysis by high-performance liquid chromatography–electrospray ionization–tandem mass spectrometry (HPLC–ESI–MS-MS). HPLC–ESI–MS-MS was performed on a Thermo Fisher LTQ Orbitrap Velos Pro mass spectrometer. An Eksigent NanoLC-Ultra 2-D HPLC system was used, with separation accomplished with a PicoFrit column (New Objective; 75-mm inside diameter [i.d.]) packed to 15 cm with C_18_ adsorbent (Vydac; 218MS; 5 mm; 300 Å). Precursor ions were acquired on the Orbitrap at a resolution of 60,000 (*m/z* 400). Data-dependent collision-induced dissociation spectra of the six most intense ions in the survey scan were acquired from the linear trap while the precursor ion spectra were being collected. Mascot (Matrix Science, London, United Kingdom) was used to search the MS files against a locally generated SPN3US protein database that had been concatenated with the Swiss-Prot database (version 51.6). Carbamidomethylation was considered a fixed modification and methionine oxidation a variable modification; semitrypsin was specified as the proteolytic agent. Subset searching of the Mascot output with X! Tandem (The Global Proteome Machine Organization; http://www.thegpm.org/tandem/), determination of probabilities of peptide assignments and protein identifications, and cross-correlation of the Mascot and X! Tandem identifications were accomplished with Scaffold (Proteome Software), using the MudPIT option to combine the results from the six slices in each lane.

### Transmission electron microscopy (TEM).

Purified wild-type and mutant particles were adsorbed to 400-mesh carbon-coated grids and negatively stained with phosphotungstic acid (PTA), ammonium molybdate, or uranyl formate. Samples were examined at 80.0 kV using an FEI Tecnai T12 transmission electron microscope.

## RESULTS

### SPN3US shares homologous proteins with other giant phages, including ϕKZ.

**FIG 1:**
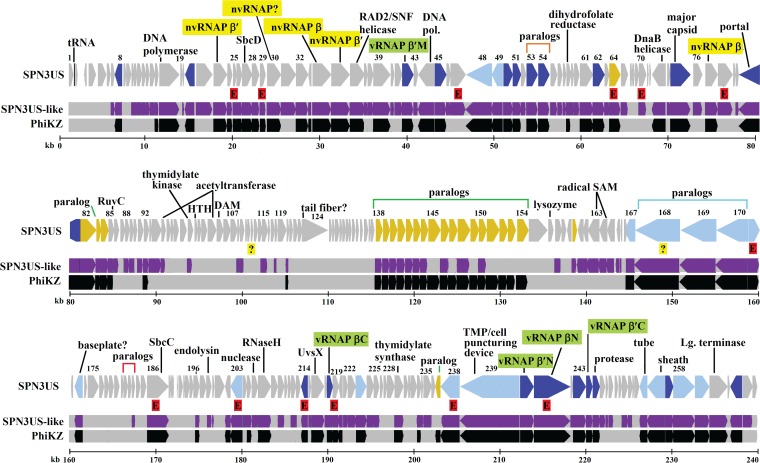
Map of the SPN3US genome highlighting genes with homologs. Homologs identified in both phages PhiEaH2 and CR5 are shown in purple and homologs of ϕKZ in black. Newly identified essential genes are indicated by red-shaded Es, and genes whose essential status requires confirmation are indicated by question marks. In the SPN3US track, genes colored dark blue have a homolog of a head protein in ϕKZ, genes colored light blue have homology with a tail protein in ϕKZ, and genes colored yellow have homology with a virion protein in ϕKZ whose head/tail location is unassigned. Subunit names of the vRNAP are shaded in green. Subunit names of the nvRNAP are shaded in yellow.

Proteins from two long-genome phages, Erwinia phage PhiEaH2 (243,050 bp) (43) and Cronobacter phage CR5 (223,989 bp) (44), were repeatedly observed to have matches to SPN3US proteins with the highest percent identity and lowest E value, as determined by PSI-BLAST, of any phage or nonphage proteins in the nr or env_nr databases. Overall a total of 215 (81%) and 156 (59%) SPN3US proteins were determined to be homologs of proteins in PhiEaH2 and CR5, respectively. CoreGenes (38, 39) confirmed that 147 of these SPN3US homologs were shared by both PhiEaH2 and CR5 (Fig. 1; see Table S1 in the supplemental material).

**FIG 2:**
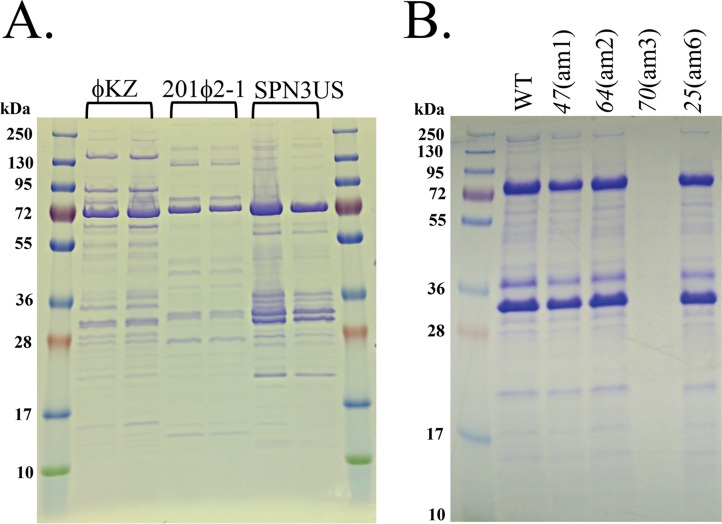
SDS-PAGE profiles of SPN3US, ϕKZ, and 201ϕ2-1 phages. (A) Profiles of CsCl step gradient-purified P. aeruginosa phage ϕKZ, P. chlororaphis phage 201ϕ2-1, and S. enterica serovar Typhimurium phage SPN3US. (B) Profiles of wild-type (WT) and amber mutant phages of SPN3US after propagation on nonpermissive S. enterica serovar Typhimurium (strain TT9079).

CoreGenes identified 69 proteins in SPN3US, PhiEaH2, and CR5 as being homologs of proteins in the phage ϕKZ (Fig. 1; see Table S1 in the supplemental material). PSI-BLAST searches identified an additional 22 SPN3US gene products as having homologs in ϕKZ (see Table S1 in the supplemental material). Among these homologs, 61 SPN3US proteins are similar to ϕKZ proteins that are part of its virion (15) (see Table S1 in the supplemental material). This number of similar virion proteins is supported by the similarity in the protein profiles of SPN3US, ϕKZ, and 201ϕ2-1 (Fig. 2). Of the SPN3US virion proteins with similarity to those in ϕKZ and other related phages, only 10 have been assigned specific functions. SPN3US and CR5 are classified as myoviruses (phages with contractile tails) in GenBank; however, the entry for PhiEaH2 (JX316028) notes that it is a siphovirus. Our search results indicate that essentially all of the structural genes of SPN3US are extremely similar to genes in PhiEaH2, including the tail sheath, indicating PhiEaH2 is clearly a myovirus.

### Isolation of SPN3US amber mutant phage candidates.

**FIG 3:**
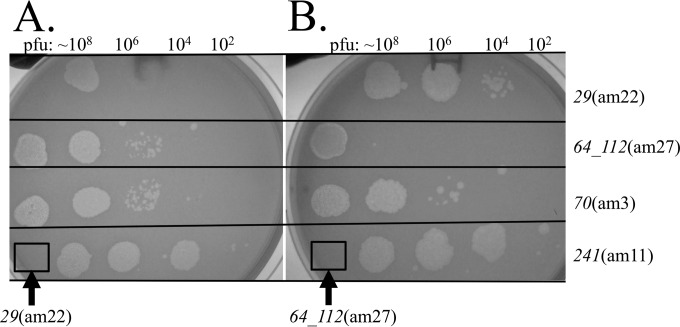
Cross-plating of SPN3US amber mutants in the nonpermissive S. enterica serovar Typhimurium strain (TT9079). Approximately 10^7^ particles of *29*(am22) (A) and *64_112*(am27) (B) were seeded into the bacterial overlay (the boxed areas show no plaques/lysis in the lawn). Mutant phages were spotted onto the overlay, with approximate numbers of PFU in each spot indicated. Note that when *29*(am22) and *64_112*(am27) were spotted onto the overlays containing themselves, there was only clearing caused by lysis from without in the presence of a large number of particles.

Mutant phage candidates were isolated from hydroxylamine-treated SPN3US and identified by growth on the permissive suppressor (*supD*) strain of Salmonella and inability to grow on nonpermissive strains. These stocks typically had titers of 10^11^ to 10^12^ PFU/ml and low reversion rates as determined by tests on two different nonpermissive hosts (see Table S2 in the supplemental material). Most of the amber mutants grew equally well on a nonsuppressor strain (TT9079) containing a plasmid-borne tRNA^Ser^ (generously supplied by David Peabody, University of New Mexico), confirming the amber identification and amino acid insertion. Most were also able to propagate on the nonpermissive strain with a plasmid-borne tRNA^Gly^, but only 23 were able to propagate in the strain with a plasmid-borne tRNA^Ala^. These plating experiments were performed to ensure that, for each mutant, the amber phenotype was reproducible on different suppressor strains. In addition, we performed cross-plating experiments with the amber mutants to enable us to select mutants for DNA sequencing that were able to rescue one another by complementation and/or recombination (Fig. 3) to reduce the likelihood of sequencing repeat mutations.

### Genome sequencing of SPN3US amber mutant phage populations.

To assay at the genome level the success of our mutant isolation, we initially sequenced a mixture of all SPN3US amber mutant phages on an Illumina HiSeq machine. This first population sequencing included all 50 mutant phage candidates mixed in approximately equal numbers prior to DNA extraction. More than 77 million sequencing reads (100-bp paired ends) were acquired for the mixture, 99.7% of which mapped to the SPN3US genome. Samtools mpileup (http://www.htslib.org/) was applied, and variants were called at an alternative allele frequency of >0.5%. Since hydroxylamine treatment causes C-to-T transition mutations, only TGG-to-TAG or CAG-to-TAG nonsense mutations were expected. Thirty-one different amber mutations were identified in annotated SPN3US coding regions (Table 1). Consistent with hydroxylamine mutagenic specificity, 25 of these mutations resulted from a mutation in a glutamine codon and 6 mutations resulted from a mutation in a tryptophan codon. The frequency of each alternate allele within the mixture ranged from 0.4 to 12.9% (Table 1). Despite the fact that every effort was made to pick only original *de novo* mutants prior to growth of the mutagenized phage population, it remains a possibility that amber mutant phages multiplied and migrated in the early top agar-containing plate to generate by growth and spreading repeat mutant plaques in the plate. Regardless of whether this was their origin, the number of different mutations identified was lower than expected. This, in conjunction with the ∼30-fold range of variant frequencies, suggested that some mutant phages might contain repeats of individual mutations (resulting in a high frequency) or that some mutations may have been missed if they had a frequency too low to be identified or occurred in a coding region that was not annotated. Further sequencing confirmed that repeat mutations did exist among the mutants and also that low-frequency mutations could be missed (see below).

**TABLE 1:** Identification of mutations resulting in amber codons in the SPN3US genome in a mixture of 50 mutant candidates^*a*^

Genome position^*b*^	Gene location	Reference base	Alternate base	No. for reference base^*c*^	No. for alternate base^*d*^	Alternate frequency (%)
**215999**	***241***	**C**	**T**	28,278	3,645	12.9
**23427**	***29***	**C**	**T**	17,315	1,776	10.3
195232	*225*	C	T	33,887	2,181	6.4
**169242**	***186***	**C**	**T**	22,789	1,374	6.0
**186856**	***214***	**G**	**A**	22,375	1,240	5.5
37455	*39*	C	T	31,428	1,439	4.6
192318	*222*	G	A	25,506	995	3.9
33261	*35*	C	T	35,090	1,295	3.7
30811	*34*	C	T	37,702	1,370	3.6
**63849**	***64***	**C**	**T**	40,330	1,415	3.5
**46330**	***47***	**C**	**T**	32,816	1,092	3.3
201073	*235*	C	T	36,575	1,189	3.3
**66658**	***70***	**C**	**T**	28,668	916	3.2
153240	*169*	C	T	25,241	785	3.1
23982	*30*	C	T	34,630	1,054	3.0
230169	*258*	G	A	17,353	499	2.9
**13997**	***19***	**C**	**T**	24,577	597	2.4
**203975**	***238***	**G**	**A**	21,902	513	2.3
159924	*171*	C	T	18,225	420	2.3
**20159**	***25***	**C**	**T**	28,444	612	2.2
**190698**	***219***	**C**	**T**	34,501	731	2.1
**146047**	***168***	**C**	**T**	31,761	633	2.0
**159099**	***171***	**C**	**T**	48,284	898	1.9
**63957**	***64***	**C**	**T**	41,325	638	1.5
170989	*186*	G	A	30,758	416	1.4
**179577**	***203***	**C**	**T**	26,162	322	1.2
**179287**	***203***	**G**	**A**	29,504	352	1.2
**77022**	***78***	**C**	**T**	33,542	239	0.7
81829	*82*	G	A	37,167	219	0.6
229576	*257*	C	T	33,223	183	0.6
**170544**	***186***	**C**	**T**	30,232	126	0.4

a Mutations subsequently identified in individual mutant phage genomes (Tables 2 and 3) are in boldface.

b Position relative to that of GenBank accession number JN641803.1.

c Number of times reference base observed.

d Number of times alternate base observed.

We decided to test the effectiveness of sequencing just two amber mutant candidates mixed in amounts that varied by a factor of 6.25, as theoretically such an approach would be more economical. These mixtures were sequenced on an Illumina HiSeq (150-bp paired-end reads), and SNPs were identified using SeqManPro (Table 2). Four amber mutations were identified using this approach, and their presence in each phage was confirmed by Sanger sequencing of the region of interest after PCR amplification. Sanger sequencing was performed by Genewiz Inc. These four mutation sites had been identified in the mixture of 50 amber mutants. In each double mixture, the frequency (percentage) of each SNP causing an amber mutation was close to that expected based on the numbers of particles added to each mixture. However, for other mutation sites (such as synonymous and nonsynonymous missense sites), it was demonstrated that there was a greater range in SNP frequencies, resulting in difficulty in concluding with certainty to which mutant these nonamber mutations belonged. These data also indicated that for some mutants there could be a considerable number of nonamber mutations; for instance, a total of 115 mutations were detected in mixture 2, which could potentially have an impact on the phenotype of the mutant. This led us to conclude that for further studies on mutants it would be desirable to have their genomes individually sequenced to ensure that all the mutations in each mutant are clearly documented.

**TABLE 2:** Amber mutation sites in mixtures containing two SPN3US amber mutants whose particles were combined in amounts differing by 6.25-fold

Mixture	Mutant	Mutation position^*a*^	SNP %	SPN3US ORF	DNA change^*b*^	Amino acid change^*c*^
1	am21	186856	85.70	*214*	c.682C→T	p.Q228.
	am28	203975	13.80	*238*	c.1186C→T	p.Q396.
2	am24	159099	82.30	*171*	c.361C→T	p.Q121.
	am30	190698	15.70	*219*	c.271C→T	p.Q91.

a Position relative to that of GenBank accession number JN641803.1. Amber mutation positions were confirmed in each mutant by Sanger sequencing.

b “c.” indicates the coordinate of the mutation within the open reading frame and its reference to called base change.

c Periods after position numbers indicate that the glutamine at that position in the polypeptide chain is not replaced, as its codon has been mutated to a nonsense codon and the protein product truncated.

### Genome sequencing of individual SPN3US amber mutant phages.

Fourteen individual SPN3US mutant genomes were barcoded and underwent genome sequencing. Three genomes (am1, am6, and am26) were sequenced using HiSeq technology (Table 3). Each of the genomes had excessive depth of coverage, and it was deduced that it would be more appropriate to sequence individual genomes using MiSeq technology, and subsequently, 11 mutant candidates were sequenced with this technology (see Table S3 in the supplemental material). In the 14 genomes, 15 different amber mutations were detected in SPN3US coding regions at a SNP percentage of 99.5% or higher. Thirteen of these amber mutation positions had been identified in the mixture of 50 mutants, with the same mutation position detected in 2 mutants (am11 and am43), supporting our expectation of repeat mutations. Twelve mutants had a single amber mutation per genome, making the classification of the genes in which they occurred as “essential” straightforward (Table 4).

**TABLE 3:** Amber mutations identified in SPN3US individual mutant phage genomes

Mutant	Genome reference position^*a*^	Reference base	Called base	SNP %	SPN3US ORF	DNA change^*c*^	Amino acid change^*d*^
am1^*b*^	46630	C	T	99.8	*47*	c.1432C→T	p.Q478.
am2	63849	C	T	99.6	*64*	c.763C→T	p.Q255.
am3	77022	C	T	99.6	*78*	c.1459C→T	p.Q487.
am6^*b*^	20159	C	T	99.5	*25*	c.184C→T	p.Q62.
am11	215999	C	T	100.0	*241*	c.2011C→T	p.Q671.
am13	66658	C	T	99.7	*70*	c.181C→T	p.Q61.
am18	179577	C	T	99.8	*203*	c.377G→A	p.W126.
am19	179287	G	A	99.8	*203*	c.667C→T	p.Q223.
am22	23427	C	T	100.0	*29*	c.358C→T	p.Q120.
am26^*b*^	13997	C	T	99.5	*19*	c.70C→T	p.Q24.
	146047	C	T	99.8	*168*	c.4673G→A	p.W1558.
am27	63957	C	T	99.5	*64*	c.871C→T	p.Q291.
	100963	C	T	99.9	*112*	c.100C→T	p.Q34.
am39	169242	C	T	99.5	*186*	c.343C→T	p.Q115.
am43	215999	C	T	100.0	*241*	c.2011C→T	p.Q671.
am50	170544	C	T	99.5	*186*	c.1645C→T	p.Q549.

a Position relative to GenBank accession number JN641803.1.

b Mutant sequenced on an Illumina Hi-Seq machine.

c “c.” indicates the coordinate of the mutation within the open reading frame and its reference to called base change.

d Periods after position numbers indicate that the glutamine in that position in the polypeptide chain is not replaced, as its codon has been mutated to an amber stop codon and the protein product truncated.

**TABLE 4:** Features of SPN3US proteins encoded by essential genes

Gene product	Mass (kDa)	Essential^*a*^	ϕKZ homolog	Function/comment^*b*^
19	10.6	ND		
25	14.6	Yes	ORF62	STR; low-copy-number tail protein
29	24.5	Yes	ORF67	
47	62.8	Yes		STR; head/neck protein
64	48.9	Yes	ORF101	STR; head, possible neck protein
70	32.2	Yes		
78	60.3	Yes		
112	10.9	ND		
168	188.1	Expected	ORF145	STR; baseplate/fiber protein
171	47.1	Yes	ORF130	STR; possible baseplate/fiber protein
186	95.7	Yes	ORF165	SbcC subunit
203	51.9	Yes	ORF157	STR; tail protein
214	28.1	Yes	ORF153	STR; head protein
219	28.6	Yes	ORF147	
238	82.1	Yes	ORF182	STR; tail protein, possible host membrane-targeting function (predicted N-terminal transmembrane domain residues 152 and 174)
241	159.1	Yes	ORF178	STR; vRNAP βN

a ND, the “essential” status of proteins encoded by genes with amber mutations in double mutants was not determined.

b STR, protein detected as part of the virion by mass spectrometry.

Overall, eight genes of the 12 individually sequenced mutants were identified as being essential. This is because for three genes there were two mutants in which a single amber mutation was identified, but at a different base pair. The genes in which there were two mutation sites were *64* (am2 and am27), *186* (am39 and am50), and *203* (am18 and am19). This is not surprising, as the mutagenesis is random (41) and the probability of a mutation occurring in a particular gene increases with increased gene length. Gene *64* is 1,314 bp, *186* is 2,511 bp, and *203* is 1,380 bp. Cross-plating of each pair of intragenic mutants under nonpermissive conditions was successful and showed that the recombination frequency of SPN3US is proportional to the distance between mutations (data not shown).

Two mutant phages, am26 and am27, each had two amber mutations in their respective genomes. The amber mutation in *112* of am27 was not detected previously in the sequencing of the mutant population, although the amber mutation in *64* had been, raising the possibility of additional undetected amber mutations in the mutant population. Since *64* had an individual amber mutation in am2, it was assigned as essential. The assignment of the three other genes mutated in double mutants, *19*, *112*, and *168*, is less straightforward, as one or both mutations in each genome could feasibly be essential.

Of the 20 amber mutations identified, only two (in *203* and *168* in am18 and am27, respectively) resulted from the mutation of a tryptophan codon; the rest resulted from the mutation of a glutamine codon. To ensure there were no unusual codon usage patterns within the SPN3US genome causing tryptophan codons to be rarer than glutamine codons, we determined the codon usage of the entire genome and of the individual genes mutated in this study using the Countcodon software (available at http://www.kazusa.or.jp/codon/countcodon.html). These analyses showed there are similar total numbers of Gln-CAG (1,398) and Trp-UGG (1,441) codons throughout the SPN3US genome and revealed no special biases for these codons within the mutated genes (data not shown). It is possible that our observation of nearly 10-fold more mutated glutamine than tryptophan codons in the mutants could be due to the relatively small number of mutants sequenced. Alternatively, it might be an unintentional bias resulting from our use of a serine suppressor strain and the fact that serine replaces glutamine more effectively than tryptophan with its large aromatic side chain. It will be interesting to determine if this pattern holds by the sequencing of more mutants and to test it further by using glutamine and tryptophan suppressor strains for future mutant isolations.

### Newly identified SPN3US essential genes.

Thirteen SPN3US genes were demonstrated to be essential in this study (Table 4). Of these, only two genes encoded proteins with previously assigned specific functions, gp186 and gp241. gp186 is a predicted SbcC subunit of an SbcCD complex (35), and gp241 is the β subunit of a predicted vRNAP subunit. In addition to gp241, six other SPN3US essential genes, *25*, *64*, *171*, *203*, *214*, and *238*, were predicted to encode proteins that were part of the virion based on their similarity to proteins in ϕKZ that had been identified as virion proteins. While it has not been conclusively demonstrated that *168* is an essential gene, we have designated this gene “expected” essential, as it is a member of a paralog family that is found in each member of the Pseudomonas phages that are related to ϕKZ. gp168 is similar to ϕKZ gp131, which was demonstrated to be located at the periphery of the baseplate and also possibly associates with fibers that emanate from the baseplate by immune-gold labeling (12).

### Proteomes of phage particles resulting from mutations in SPN3US *241* and *64*.

**FIG 4:**
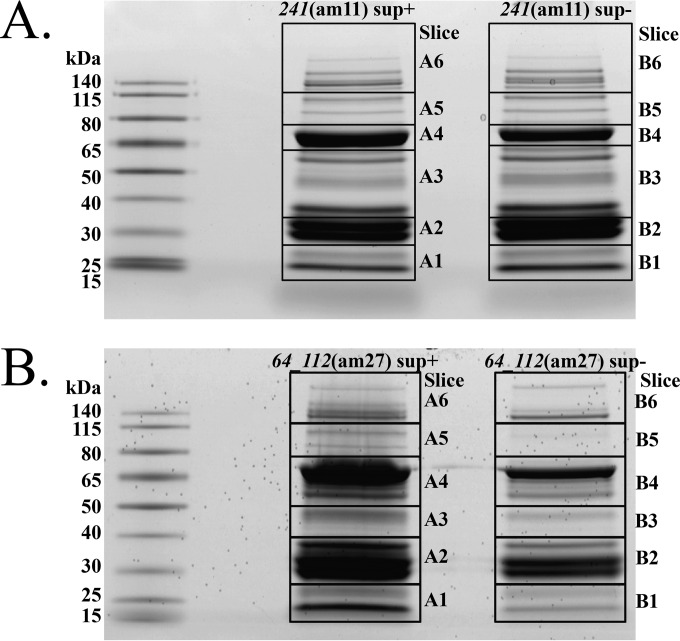
SDS-PAGE gels used for mass spectrometry of SPN3US amber mutants. Shown are *241*(am11) (A) and *64_112*(am27) (B). sup+, mutant propagation on the permissive host; sup−, mutant propagation on the nonpermissive host.

Two mutants, *241*(am11) and *64_112*(am27), with mutations in the genes encoding well-conserved and predicted virion genes were selected for propagation in the nonpermissive and permissive host strains to determine the effect of knocking out their respective genes. Purified particles from both mutants grown on the permissive host purified at a buoyant density consistent with that of the wild-type phage, which is similar to that obtained for both ϕKZ and 201ϕ2-1 (1.38 g/ml). The *241*(am11) particles produced in the nonpermissive host purified at a slightly lower buoyant density (1.392 g/ml) than those produced from the permissive host (1.396 g/ml), and that, in addition to small observable differences in the protein profile of each sample (Fig. 4A, slices A6 and B6), indicated there might be differences in their proteomes that could be detected by mass spectrometry. Conversely, the particles produced by growth of *64_112*(am27) in the nonpermissive hosts had very different buoyant densities (1.4240 to 1.4298 g/ml), so it was assumed there would be a marked difference in their proteomes. Consequently, samples of *241*(am11) and *64_112*(am27) propagated on both host strains were subjected to gel separation and analysis by LC–MS-MS (GeLCMS) (Fig. 4).

**FIG 5:**
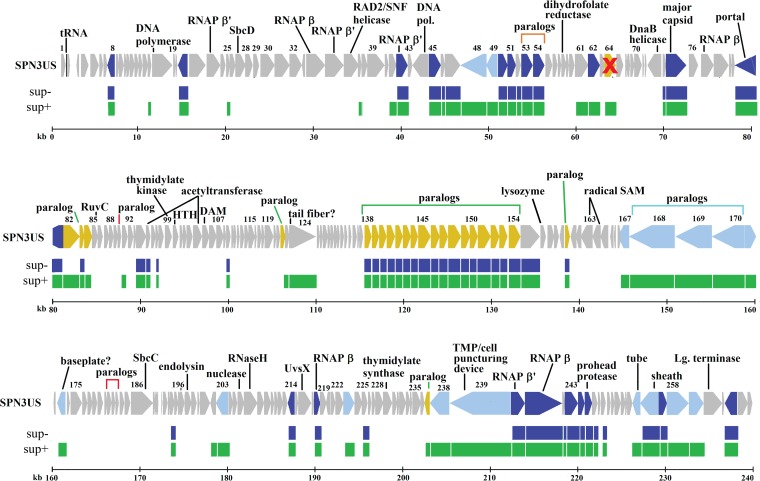
Map of SPN3US genome comparing proteomes of mutant *64_112*(am27) when grown on permissive (sup+) and nonpermissive (sup−) hosts. The proteome of *64_112*(am27) propagated on the sup+ or permissive host is indicated in green, and its proteome when propagated on the sup− or nonpermissive mutant host is indicated in blue. The gene encoding gp64 (marked with a red cross) is essential, as determined by sequencing of a mutant (am2) with a single amber mutation in its genome, in the gp64 gene. gp112 is not a virion protein, and its essential status requires clarification. In the SPN3US track, genes colored dark blue have homology with a head protein in ϕKZ, genes shaded light blue have homology with a tail protein in ϕKZ, and genes colored yellow have homology with a virion protein in ϕKZ whose head/tail location in unassigned.

Peptides from 83 different SPN3US proteins were detected by mass spectrometry in each mutant propagated on the permissive host (see Table S4 in the supplemental material). Sixty of these proteins are similar to ϕKZ virion proteins (Fig. 5), as determined by both the CoreGenes analyses and additional PSI-BLAST searches. In each mutant, it was of interest to determine whether a partial product of the mutated gene would be incorporated into the mature particle. For instance, in *241*(am11), the amber mutation in *241* would result in the truncation of the normally 1,401-residue protein at residue 670, and in *64_112*(am27), the mutation in *64* would result in a product of only 290 residues compared to the normal 437 residues. However, in each mutant, no peptides from the mutated gene product were detected. In addition, other proteins detected in particles produced from the permissive host were not detected in the particles propagated on the nonpermissive host, 7 and 28 different proteins for am11 and am27, respectively. This indicates that the incorporation of both gp241 and gp64 into the virion is essential for the incorporation of the full complement of proteins in the virion.

The fact that gp241 was not included in particles propagated on the nonpermissive host was very definitive, with no spectra detected for tryptic peptides in this large protein. This is in contrast to the 110 total spectra detected for gp241 in the sample propagated on the permissive host. In addition to gp241, six other SPN3US proteins were detected in *241*(am11) and *64*(am27) propagated in the permissive strain but were not detected when *241*(am11) was propagated on the nonpermissive strain (see Table S4 in the supplemental material). They are gp37, gp42, gp158, gp218, gp240, and gp244. Three of these proteins, gp42, gp218, and gp240, along with gp241, are all predicted to be subunits of the vRNAP (Table 5), so they are expected to be part of the phage head.

**TABLE 5:** Mass spectral counts detected for *64_112*(am27) and *241*(am11) propagated on permissive (sup+) and nonpermissive (sup−) hosts^*a*^

Gene product	Length (aa)	Total spectrum count				vRNAP subunit
		am27		am11		
		sup+	sup−	sup+	sup−	
37	127	6	0	6	0	
42	431	37	36	27	0	β′M
158	171	3	0	2	0	
218	222	23	28	24	0	βC
240	519	56	38	14	0	β′N
241	1,401	203	152	110	0	βN
244	240	12	13	10	0	β′C^*b*^

a *64_112*(am27) grown on the nonpermissive host formed tailless particles.

b gp244 was identified as a candidate for the C-terminal region of β′ in this study (see the text).

**FIG 6:**
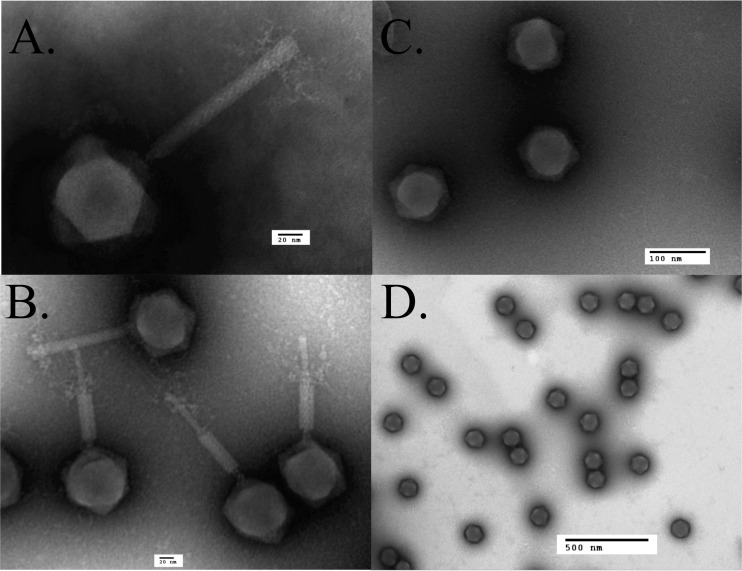
Transmission electron microscopy of SPN3US wild type and amber mutant phage *64_112*(am27). (A) Wild-type phage in a nonpurified lysate stained with PTA. (B) CsCl-purified wild-type phage stained with ammonium molybdate. (C and D) CsCl gradient-purified particles of *64_112*(am27) grown on the nonpermissive host stained with ammonium molybdate (C) and uranyl formate (D).

The gp64 gene encodes a low-abundance protein found in the SPN3US virion, as determined by the detection of only nine mass spectra in the sample from the *64_112*(am27) particles propagated on the permissive host. This is consistent with the number of spectra identified for gp64 in *241*(am11) grown on both hosts. Previous mass spectral studies also showed the homologous protein in both ϕKZ and 201ϕ2-1 to be low-abundance proteins in their respective virions (15, 17). Notably, in addition to no mass spectra being detected for gp64 in *64_112*(am27) grown on the nonpermissive host, none were identified for an additional 28 proteins normally seen in the mutant grown on the permissive host (Fig. 5; see Table S4 in the supplemental material). Among the “missing” proteins were those associated with the phage tail, such as the major tail tube protein (gp255) and the tape measure protein (gp239) (Fig. 5). Among the 52 proteins identified in the *64_112*(am27) sample propagated under nonpermissive conditions were proteins with functions associated with the head, such as the major capsid protein (gp75) and the portal protein (gp81) (Fig. 5). Examination of the gp64 samples by TEM revealed tailless particles, indicating that the proteins identified in this sample are located in the SPN3US head or neck (Fig. 6). The latter function is indicated by the fact that the heads contained DNA, implying any “plug” proteins that normally function to prevent the DNA from exiting the head after packaging had also been added to these particles. Further suggesting that the normal neck structure was nearly complete was the observance of a small number of spectra for tryptic peptides from the tail sheath protein, gp256 (8 spectra versus 686 spectra in the mutant grown on the permissive host). From these observations, we conclude that putative functions of gp64 are an aid in tail attachment to the head or a neck protein or that they have an essential role in tail assembly.

The identification of SPN3US virion proteins in the analyses of *241*(am11) and *64_112*(am27) enabled the general delineation of all the essential genes identified in this study as encoding either nonvirion or virion proteins. Three essential proteins are not associated with the virion, gp29, gp70, and gp78. gp19, whose essential status requires clarification, is also not associated with the virion. No functions can be assigned to any of these proteins based on sequence-based searches, and further investigations will be required to resolve their roles. It is interesting that several genes encoding essential proteins were determined to be located close to an expected essential gene, suggesting there may be clustering of essential genes throughout the genome. For instance, the gp78 gene is located immediately downstream of the gene encoding the N-terminal fragment of the nvRNAP β (*77*). We hypothesize that gp78 may have a regulatory function, as apparently many proteins are not produced when this protein is knocked out, as determined by the lack of detectable virion proteins being produced when *78*(am3) is propagated under nonpermissive conditions (Fig. 3B). In addition to the two head proteins, gp64 and gp241, the proteome analyses facilitated the delineation of the products of six other newly identified SPN3US essential genes into those encoding tail-associated (gp25, gp171, gp203, and gp238) and head-associated (gp47 and gp214) proteins. gp168, whose gene's amber mutation was detected in a double mutant, is also a tail protein.

## DISCUSSION

### SPN3US, a candidate type phage of a new giant phage genus, “*SPN3USlikevirus*.”

SPN3US was determined to share 55.7% of its proteins with easily identifiable homologs in both Erwinia phage PhiEaH2 and Cronobacter phage CR5 by both the CoreGenes program and PSI-BLAST. This set of homologs in these phages is comparable to the percentage shared by ϕKZ, ϕPA3, and 201ϕ2-1 (members of the genus *PhiKZlikevirus*), and according to the recommendations of Lavigne et al. (45), SPN3US, PhiEaH2, and CR5 could be considered a genus, which we refer to as “*SPN3USlikevirus*.” The fact that SPN3US shares 26% of its proteins with identifiable homologs in Pseudomonas phage ϕKZ, as determined by CoreGenes, and 37.8% when PSI-BLAST matches are included, also leads us to suggest that SPN3US could be considered a diverged member of a tentative “ϕKZ-related” phage subfamily. This conclusion is based on the taxonomic status of phages EL and OBP. It was recommended that EL be considered a separate genus within a common family based on a smaller number of easily identified homologs (30% of EL's proteins were found to be similar to those in ϕKZ). The addition of OBP to the EL-like genus enabled an extensive tabulation of more diverged ϕKZ homologs (22). It also enabled the determination that, while global synteny is disrupted by a series of inversions in the genomes of phages in this subfamily, local synteny confirmed the orthologous status of most ϕKZ homologs (22). This finding made it clear that a group of phages related to ϕKZ is supported by vertical descent of a large number of genes, which include those specifying a characteristic head and tail morphology, as well as a characteristic gene expression and replication strategy (22).

The existence of homologs of ϕKZ in an expanding group of giant phages was the incentive to study a representative by using genetics, which would first enable us to test if there was a relationship between vertically descended genes and essential genes in these phages and, second, enable the study of currently functionally unassigned proteins. We isolated amber mutants of SPN3US, the first such mutants for these giant phages. Initial sequencing of a mixture of the candidates showed the presence of amber mutations in 33 SPN3US genes, 24 with homologs in ϕKZ. Numbers of these 24 genes would also be expected to be essential based on studies of other phages; for instance, the SbcC subunit of T4 was demonstrated to be essential using genetics, and the subunits of the ϕKZ multisubunit RNAPs would be expected to be essential based on the replication of the phage in the presence of rifampin (19). However, to confirm that any of the detected SPN3US amber mutations were actually in an essential gene, each mutation must be identified as the sole amber mutation in an individual genome. Toward this goal, we sequenced 18 individual mutant phage genomes, 2 of which had double amber mutations and 16 of which had a single amber mutation per genome, enabling us to identify 13 essential genes in SPN3US.

Of the 13 SPN3US genes newly classified as essential, 9 (*25*, *78*, *171*, *186*, *203*, *214*, *219*, *238*, and *241*) were also determined to have homologs in ϕKZ, 201ϕ2-1, ϕPA3, EL, and OBP (Table 6), as does the “expected” essential tail gene, *168*. Further searches showed that the 10 proteins encoded by these genes in SPN3US also have homologs in other recently identified ϕKZ-related phages, such as Vibrio phage JM2012 (25) and Erwinia phage Ea35-70 (46), and other giant phages (Table 6), supporting the broader relevance of utilizing a genetic system to study SPN3US. Examples of several proteins well conserved in all myoviruses (major head, sheath, and terminase proteins [47]) are also included in Table 6 to illustrate the intrinsically divergent nature of homologous proteins in these phages. The homologs in the table also highlight a dichotomy in the literature as to what is referred to as a “ϕKZ-related” phage. The B. subtilis phage AR9 was recently described as belonging to the ϕKZ-related phage group (24). As illustrated in Table 6, while AR9 has homologs of several SPN3US essential genes, such as the SbcC subunit, terminase, and vRNAP subunits, there is clearly an absence of easily detectable homologs in AR9 of other essential proteins in SPN3US, which have counterparts in ϕKZ and most other phages described as being ϕKZ related in the literature. Notably, there is no easily identifiable homolog in AR9 of two highly abundant virion proteins, the major capsid and tail sheath, in SPN3US or ϕKZ, although obviously AR9 is a myovirus and has these proteins. These differences highlight a need for clearer definitions of a taxon for giant phages. Ideally, such definitions would incorporate both predicted core genes and experimentally identified essential genes, now that we have a genetic system to prove that status. The differences among these giant phages also highlight exciting and likely complex evolutionary pathways that require unraveling.

**TABLE 6:** Proteins in giant phages with similarity to SPN3US proteins that are encoded by genes identified as having an amber mutation in this study or expected to be essential

Phage	% identity of match to SPN3US protein^*a*^:																			
	gp75^*b*^	gp256^*b*^	gp260^*b*^	gp19	gp25	gp29	gp47	gp64	gp70	gp78	gp112	gp168	gp171	gp186	gp203	gp214	gp219	gp238	gp241	gp244^*c*^
PhiEaH2	88	85	87		69	78	56	71	29	49	77	44	81	63	80	78	65	75	78	88
CR5	63	67	70		51	42	27	38		26		31	46	43	57	47	44	53	59	71
ϕKZ	24	26	34		20			24				16	20	22	30	21	24	22	29	30
201ϕ2-1	23	25	34		21			23				10	23	24	29	14	23	21	34	28
ϕPA3	23	27	33		15			25				15	20	23	30	14	22	21	31	29
Ea35-70	28	26	34		24		25	23				16	22	35	25		22	22	30	30
PhiEaH1	22	27	31		17		26	23				15	20	22	29	20	17	24	30	27
RSL2	23	28	31		18			21				16	16	24	24	13	22	25	32	30
JM-2012	22	25	31		17			22				14	?	24	23	18	22	20	28	26
VP4B	21	21	28		20			22				12	20	22	24		18	17	26	24
OBP	23	20	29		22			25				13	19	22	24		18	20	26	18
EL	21	22	25		26			25				13	18	21	24		18	18	25	16
ϕR1-37	28																	25	21	17
AR9	28													13				23	21	15
BpSp	26													14				24	20	19

a Similarity was determined using PSI-BLAST with a maximum of three iterations.

b Three proteins were included as positive controls, as they must be essential in all myoviruses and are expected to be detectable in related phages. They are gp75 (major capsid), gp256 (sheath protein), and gp260 (terminase).

c The newly identified candidate vRNAP subunit, gp244, in SPN3US and its homologs are also included.

The identification of 13 essential SPN3US gene products emphasizes the potential of the SPN3US genetic system for protein function determination, as only two proteins, gp186 and gp241, previously had assigned functions based on sequence similarity. However, even for these two proteins, there is a lack of specific knowledge pertaining to their functions and interactions with other proteins, indicating the potential of their mutants. For instance, the SPN3US gp186 gene encodes a putative SbcC subunit. In Escherichia coli, SbcCD has roles in DNA repair and replication via its ATP-dependent double-stranded DNA exonuclease activity and ATP-independent single-stranded DNA endonuclease activity. The SbcC subunit is larger (1,048 amino acids [aa]), with ATPase activity, whereas the smaller (400-aa) SbcD subunit has nuclease activity. E. coli SbcC belongs to the structural maintenance of chromosomes (SMC) family, whose members are highly conserved in bacteria and have structural homology with the eukaryotic Mre11/Rad50 proteins that function to repair breaks in dsDNA (48). In light of this conservation, it is not surprising that there are homologs of the SbcCD proteins in ϕKZ and other giant phages, and as has now been shown for SPN3US gp186 and inferred for gp27 (the SbcD subunit), these proteins are also essential. In E. coli, the SbcD gene is upstream of that encoding SbcC in the *sbcDC* operon (48). Similarly, the T4 phage homolog gp46 and gp47 genes are clustered, although separated by two small hypothetical ORFs, gp46.1 and gp46.2. In contrast, in SPN3US (Fig. 1) and in ϕKZ and other related phages, the SbcC and SbcD genes have been drastically separated within the genome. This splitting of functionally grouped genes is a feature of the genomes of phages related to ϕKZ, even for genes typically grouped together in a phage genome (such as the morphogenesis genes [e.g., head-related genes]), and is suggestive of unusual and interesting evolutionary mechanisms.

The SbcCD complex of SPN3US likely has a role similar to that of T4 gp46 and gp47. Mutations in either of the T4 genes encoding these proteins result in deficiencies in recombination and DNA replication and no host DNA degradation (28). However, the similarities and differences in the roles of this complex in SPN3US require confirmation, as it is extremely divergent from that in T4—there is no sequence similarity between the proteins of the two phages that can be detected by a simple BLASTP search. The SPN3US SbcC protein is larger (95.7 kDa) than that of T4 (63.6 kDa), also suggesting potential functional differences between the two proteins. As in E. coli, both phage SbcC proteins have ATP-binding cassette domains split into an N-terminal domain containing a Walker A motif and a C-terminal domain containing an ABC transporter signature motif, Walker B motif, D loop, and H loop/switch region. However, the region between the split domains in SPN3US gp186 is more than 600 residues, approximately 300 residues longer than that of T4 gp46. In T4, the gp46-gp47 complex was determined to be a membrane protein and was suggested to be anchored to the membrane by gp47.1 by Miller et al. (28). T4 gp47.1 is a small protein (46 aa) with a predicted transmembrane domain (28). The gene immediately downstream of the SPN3US SbcC gene, the gp187 gene, encodes a small (63-amino-acid) protein with a transmembrane domain predicted by TMHMM (49), so it will be of interest to determine the other proteins with which the SPN3US SbcCD complex interacts and its cellular location.

### Using mutant proteomes to resolve the structure and assembly of a complex virion.

Two-thirds of the newly identified SPN3US essential genes encode predicted virion proteins, which is consistent with a significant proportion of the genome encoding virion proteins (∼46%), although what percentage of this is essential remains to be determined. Our examination of the proteomes of two mutants [*241*(am11) and *64_112*(am27)] with knockouts in virion protein genes was unexpectedly fruitful. The *64_112*(am27) mutant was selected for proteome analysis because the product of its mutated essential gene has homologs in ϕKZ and related phages (Table 6). In addition, the gene encoding gp64 is situated at the end of a module of apparently functionally clustered genes in these phages. This is in striking contrast to the many other genes, such as the SbcCD and RNAP genes discussed above, that are split and have undergone genomic rearrangement. This gene region is important in ϕKZ, as four of its genes encode abundant internal head proteins, and hence, it was named the IB region (15). The ϕKZ homolog of SPN3US gp64, gp101, was not identified in the head; however, the genetic basis for the *ts*13 mutant upon which that study was based is unknown.

Fortuitously, the SPN3US *64_112*(am27) mutant produced tailless particles, and based on these particles containing packaged DNA, we inferred that the heads were likely mostly complete and that gp64 is potentially a neck protein. Importantly, the purification of these particles enabled the delineation of the more than 80 proteins associated with the SPN3US particle into general categories of head (53 proteins) and tail (28 proteins). Clearly, the exact composition of each substructure within the SPN3US virion will need further investigation, but our strategy of applying proteomic and structural studies to additional virion gene mutants will facilitate the piecing together of this complex structural jigsaw. An unexpected finding was that a large, 17-member paralog family (gp138 to gp154) was identified as being part of the head, potentially the largest family of paralogs identified in any single phage genome.

Comparison of the number of different SPN3US head proteins (∼53) to the number in the T4 phage head (10) highlights the extent of the challenge in assigning functions to virion proteins and the importance of the mutants in doing so. Excitingly, such analyses will have broader relevance to the ϕKZ structural-homology group, as we determined that 60 SPN3US virion proteins are similar to ϕKZ virion proteins (Fig. 4) by CoreGenes and/or PSI-BLAST searches.

### Multimerization of the vRNAP prior to incorporation into the phage head.

gp241 is the second SPN3US essential gene identified in this study with a known predicted function, as a subunit of a multisubunit RNAP, prior to the studies. Two multisubunit RNAPs are considered hallmark features of phages related to ϕKZ, with every member containing two sets of subunits (19), one set presumably arising from a gene duplication event(s) after becoming phage borne. This is quite remarkable considering that the two subunits (β and β′) themselves are hypothesized to have arisen from an ancient duplication event (50). The phage β and β′ subunits have extremely divergent homologies with prokaryotic RNAPs, as if they have been evolving independently from cellular RNAPs for a considerable time. The recent identification of homologous subunits in the phage AR9 (24) supports this, as the presence of the RNAP genes in phages infecting both Gram-positive and Gram-negative hosts suggests that if these genes underwent little horizontal exchange they may have descended from an ancestral phage prior to the Gram-positive and Gram-negative split, estimated to have occurred over 3 billion years ago (51).

**FIG 7:**
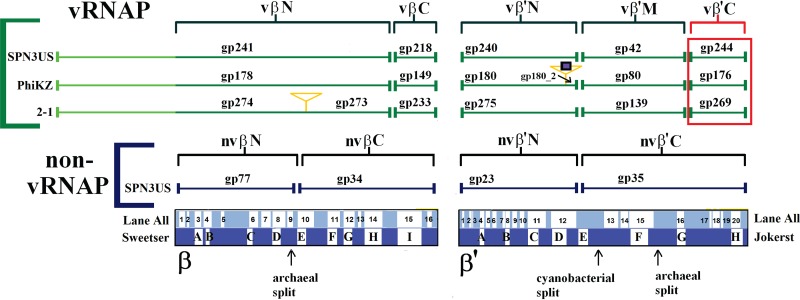
Scheme showing the homologous vRNAP subunits of SPN3US, ϕKZ, and 201ϕ2-1 (2-1). Subunits detected by mass spectrometry in purified virions are bracketed in green. The newly identified C-terminal subunit of vRNAP β′ (SPN3US gp244) is boxed in red. The orange triangles indicate introns, and the blue square indicates a homing nuclease. The SPN3US nvRNAP subunits are also included. Cellular RNAP conserved regions and archaeal and cyanobacterial subunit split sites in β and β′ are indicated (56, 61). Note that the ∼300-residue lineage-specific insert in T. thermophilus β′ located between the Lane all regions a5 and a6 is not indicated.

Remarkably, in ϕKZ, one of the RNAPs, the vRNAP, is packaged into the phage head among the densely packaged DNA (15), and in this study, we have confirmed that the SPN3US vRNAP is head associated, as it was identified in the tailless mutant. In the SPN3US complex, gp241 represents the N-terminal fragment of the β subunit and gp218 represents a smaller, C-terminal fragment (Fig. 7). The other predicted subunits of the SPN3US vRNAP are gp240 and gp42, which represent the N- and C-terminal subunits of the β′ subunit, respectively. The absence of all vRNAP subunits in the proteome of *241*(am11) grown under nonpermissive conditions indicates that gp241 has a crucial role in the incorporation of the other subunits into the phage head. The enzyme complex likely assembles prior to its incorporation into the head, and we hypothesize that the N-terminal domain of gp241 targets the complex into the head, as it has a long N-terminal domain (∼400 residues) that has no homology with prokaryotic RNAP β. These findings provide further support for the notion that the vRNAP of SPN3US (and, we infer, related phage vRNAPs) has extremely unique features, including the following: (i) the enzyme assembles from gene products whose transcripts are from very different locales in the genome; (ii) the vRNAP subunits apparently assemble/multimerize prior to incorporation into the head; (iii) the vRNAP subunits must transition from within the head through the connector complex and tail tube (the tube diameter is 4.5 nm in ϕKZ [10]) into the host cell, which hints that the proteins cannot be in a native form to do this; and (iv) the subunits must reassemble (based on point iii) into a functional complex once in the host cell. Additionally, the state of the vRNAP within the mature head is completely unknown, but based on the fact that the DNA concentration in tailed phage heads is typically about 500 mg/ml (52, 53), it is likely that structurally the vRNAP is very different from that of its active form or that of any active prokaryotic RNAP. It has been proposed that the high-force packaging enzyme compresses native proteins within the prohead into their mature head state, thereby allowing exit through the narrow-diameter portal and tail tube (54).

### Finding a “missing” vRNAP subunit.

The SPN3US proteins gp241, gp218, gp240, and gp42 and their homologs in other giant phages that were previously identified as forming the vRNAP complex (19) account for all of the highly conserved regions in prokaryotic RNAP, with the exception of several hundred amino acids in the C-terminal region of β′ (approximately residues 1260 to 1524 of the RNAP β′ of Thermus thermophilus HB8). The C-terminal region of β′ was initially recognized as being conserved in both prokaryotic and eukaryotic RNAPs by Jokerst et al. and designated region H (55). Due to the massive increase in sequence data deposited in the databases, Lane and Darst clarified the conserved regions and lineage-specific domain insertions in the β and β′ subunits of eukaryotes, eukaryotic viruses, bacteria, and archaea (56). They identified four motifs (a17 to a20) at the C terminus of the β′ subunit that are conserved in all RNAPs, with a20 equating to the previously defined region H (56). Structurally, this region has an important role in the clamp, and a20 in particular serves as a hinge to mediate clamp movement (57).

There has been no identified counterpart to region β′ a17 to a20 in any ϕKZ-related phage vRNAP, although a counterpart to the region is present at the C terminus of the nvRNAP β′ subunit, SPN3US gp35 (Fig. 7). Of all the conserved regions in β and β′ of prokaryotic and eukaryotic RNAPs, β′ a17 to a20 is the most weakly conserved (58). In addition, between different bacterial taxa, there is considerable variation in β′ a17 to a20, as the region is associated with two sites in which domains of various lengths have been inserted: β′In6, which occurs immediately after a16, and β′In7, which occurs between a19 and a20 (56). Based on the variability in this region, if a subunit containing the region existed for the phage vRNAP, it would not be surprising that it was not identified, especially if it had been split into a separate polypeptide. This is especially so because of the high level of divergence of the other phage RNAP subunits from prokaryotic RNAPs, even those subunits that contain regions traditionally more conserved in prokaryotic RNAPs, such as SPN3US gp240, which contains β′ a12 (the region containing the catalytic motif and centered in the double-psi beta barrel [DPBB], the most highly conserved region and structure in all RNAPs) and shares only 14% percent identity with the closest prokaryotic β′ subunit, as determined by BLASTP.

If region a17 to a20 of β′ is essential for functionality of the phage vRNAP, and if such a subunit exists, it could be hypothesized that that subunit would incorporated with the other subunits into the head. Therefore, any protein normally present in the SPN3US virion but absent in the gp241^−^ mutant would be a candidate for the missing region of the β′ subunit. Three proteins, gp37, gp158, and gp244, meet this criterion; however, gp37 and gp158 were determined not to be components of the phage head, as they were not identified in tailless particles (Table 5) (see below). In contrast, gp244 was identified in the SPN3US heads. gp244 is 240 residues in length, making it the most appropriate length for the missing region of β′ of the three functionally unassigned proteins not detected in the gp241^−^ mutant. gp244 has a homolog in all other giant phages with a vRNAP, which would be expected if it represented β′C (Table 6). In contrast, homologs of gp37 and gp158 can be detected by PSI-BLAST only in PhiEaH2 and CR5. In addition, in all the giant phages that have undergone mass spectral analyses, the homolog of gp244 has been identified as part of the virion (201ϕ2-1 [17], ϕKZ [15], EL [59], ϕR1-37 [60], and AR9 [24]).

Importantly, of the three gene products implicated in vRNAP function by the pleiotropic effect of *241*(am11), only gp244 could be assigned a specific role in the vRNAP structure through sensitive sequence similarity measures (S. C. Hardies, personal communication). The analysis followed an observation made at the time when Pseudomonas phage 201ϕ2-1 was added to the ϕKZ-like genus. HHpred style hidden Markov models (HMM) had been used to align all of the known ϕKZ-like vRNAP and nvRNAP polypeptides with cellular RNA polymerase. All of the established conserved RNAP motifs could be accounted for, with the exception that β′ motif H, defined by Jockerst et al. (55), and the corresponding C-terminal segment of the vRNAP appeared to be missing (Fig. 7). Now that SPN3US gp244 (a homolog of ϕKZ gp176 and 201ϕ2-1 gp 269) has been identified as a candidate for the missing segment, an HHpred HMM was constructed and aligned with various RNAP models. The gp244 HMM matched both an nvRNAP model and a cellular-region β′ a17-to-a20 model with E values of 0.05 (database size = 1). In both cases, the match was confined to the conserved region β′ a20. This match was seen again when an HMM based on gp42 fused with gp244 was aligned with the nvRNAP HMM (based on gp35) with an E value of 4.6E−20. The E values for the gp244-alone HMM are too weak to implicate gp244 as an RNAP homolog by themselves, but with gp244 nominated by another method, these searches confirm that gp244 aligns as expected to comprise the missing C-terminal segment of vRNAP β′ and illustrate the value of blending genetics with proteomics and bioinformatics.

### Conclusions.

Recent studies have demonstrated that extraordinarily large bacterial viruses (giant phages) are abundant in a variety of environments (9, 17, 24, 25, 35, 43). The ability to define such giant phages at the genome level has been limited due to the fact that a large proportion of their proteins have not been characterized. In this study, we demonstrated the suitability of a giant phage that infects Salmonella as a tractable model for related phages. Nucleotide sequencing of 18 mutants identified 13 essential genes, 11 of which were previously functionally unassigned. Proteomic analyses of two mutants revealed new information regarding the virion that could not have been determined without genetics, including the identification of a “missing” fifth vRNAP subunit. Further isolation and sequencing of SPN3US mutants is under way with the goal of identifying other essential head proteins to allow us to further dissect the giant phage head assembly and structure. Research utilizing the T4 model system was pivotal for the formation and development of molecular genetics and continues to produce tremendously important findings relevant to many research fields. Our novel SPN3US model system opens the door to future multidisciplinary analyses we believe will be equally illuminating in the understanding of giant phage biology.

## Supplementary Material

Supplemental material
