# Supplementary material for: Identification of Essential Genes in the Salmonella Phage SPN3US Reveals Novel Insights into Giant Phage Head Structure and Assembly
Source: J Virol. 2016 Oct 28;90(22):10284–98. doi: 10.1128/JVI.01492-16 (PMC5105663; doi:10.1128/JVI.01492-16)
Supplement: Supplemental material [file supp_90_22_10284__index.html]

Identification of Essential Genes in the Salmonella Phage SPN3US Reveals Novel Insights into Giant Phage Head Structure and Assembly — Supplemental material 

# Identification of Essential Genes in the Salmonella Phage SPN3US Reveals Novel Insights into Giant Phage Head Structure and Assembly

## Supplemental material

- Supplemental file 1 -

  Table S1 (Homologous proteins found in SPN3US, *Erwinia* phage phiEaH2, phage CR5, and *Pseudomonas* phage ϕKZ.)

  Table S2 (Titers and reversion rates of SPN3US amber mutant candidates.)

  Table S3 (Mutations detected in SPN3US amber mutants.)

  Table S4 (Mass spectral counts obtained by GelCMS for the two amber mutants, *241*am11 and *64\_112*am27.)

  PDF, 374K
