# Supplementary material for: Identification of Essential Genes in the Salmonella Phage SPN3US Reveals Novel Insights into Giant Phage Head Structure and Assembly
Source: J Virol. 2016 Oct 28;90(22):10284–98. doi: 10.1128/JVI.01492-16 (PMC5105663; doi:10.1128/JVI.01492-16)
Supplement: Supplemental material [file JVI.01492-16_zjv999182100so1.pdf]

A new model for giant phage research: Identification of essential genes in the *Salmonella* giant phage SPN3US reveals novel insights regarding phage structure and biology.

List of Supplementary Material

Table S1. Homologous proteins found in SPN3US, *Erwinia* phage phiEaH2, phage CR5 and *Pseudomonas* phage ϕKZ, as determined by CoreGenes

Table S2. Titers and reversion rates of SPN3US amber mutant candidates.

Table S3. Mutations detected in SPN3US amber mutants.

Table S4. Mass spectral counts obtained by GelCMS for the two amber mutants, *241*am11 (gp241-) and *64_112*am27 (gp64-/ gp112-), propagated on permissive (sup+) and non-permissive (sup-) strains of *Salmonella* Typhimurium (TT6675 and TT9079, respectively).

Table S1. Homologous proteins found in SPN3US, *Erwinia* phage PhiEaH2, phage CR5 and *Pseudomonas* phage ϕKZ as determined by CoreGenes (http://binf.gmu.edu:8080/CoreGenes3.5/BatchCoreGenes.html). **STR.**, refers to protein identified as part of the virion.

SPN3US	SPN3US	PhiEaH2	CR5	ϕKZ	phiKZ (additional homologs detected by PsiBlast)
**gp**	**JN641803**	**JX316028**	**JX094500**	**NC_004629**	**NC_004629**
7	GI:349502718 PRODUCT:hypothetical protein	GI:402761512 PRODUCT:hypothetical protein	GI:398256211 PRODUCT:hypothetical protein		
8	GI:349502719 PRODUCT:hypothetical protein	GI:402761513 PRODUCT:hypothetical protein	GI:398256212 PRODUCT:hypothetical protein	GI:29134978 PRODUCT:ORF042 **STR.**	
11	GI:349502722 PRODUCT:hypothetical protein	GI:402761514 PRODUCT:hypothetical protein	GI:398256215 PRODUCT:hypothetical protein		
13	GI:349502724 PRODUCT:hypothetical protein	GI:402761516 PRODUCT:hypothetical protein	GI:398256217 PRODUCT:hypothetical protein		
14	GI:349502725 PRODUCT:hypothetical protein	GI:402761517 PRODUCT:hypothetical protein	GI:398256218 PRODUCT:hypothetical protein		
16	GI:349502727 PRODUCT:hypothetical protein	GI:402761519 PRODUCT:hypothetical protein	GI:398256220 PRODUCT:hypothetical protein		
17	GI:349502728 PRODUCT:hypothetical protein	GI:402761520 PRODUCT:hypothetical protein	GI:398256221 PRODUCT:hypothetical protein		GI:29134985 PRODUCT:ORF049 **STR.**
18	GI:349502729 PRODUCT:hypothetical protein	GI:402761521 PRODUCT:hypothetical protein	GI:398256224 PRODUCT:hypothetical protein	GI:29134986 PRODUCT:ORF050	
20	GI:349502731 PRODUCT:hypothetical protein	GI:402761522 PRODUCT:hypothetical protein	GI:398256313 PRODUCT:hypothetical protein		

21	GI:349502732 PRODUCT:hypothetical protein	GI:402761524 PRODUCT:hypothetical protein	GI:398256226 PRODUCT:hypothetical protein	GI:29134988 PRODUCT:ORF052 **STR.**	
22	GI:349502733 PRODUCT:hypothetical protein	GI:402761525 PRODUCT:hypothetical protein	GI:398256227 PRODUCT:hypothetical protein		
23	GI:349502734 PRODUCT:putative DNA-directed RNA polymerase beta subunit 1	GI:402761526 PRODUCT:putative PHAGE DNA-directed RNA polymerase beta subunit 3	GI:398256228 PRODUCT:putative DNA-directed RNA polymerase beta' subunit	GI:29134991 PRODUCT:ORF055	
24	GI:349502735 PRODUCT:hypothetical protein	GI:402761527 PRODUCT:hypothetical protein	GI:398256229 PRODUCT:hypothetical protein	GI:29134995 PRODUCT:ORF059	
25	GI:349502736 PRODUCT:hypothetical protein	GI:402761528 PRODUCT:hypothetical protein	GI:398256230 PRODUCT:hypothetical protein	GI:29134998 PRODUCT:ORF062 **STR.**	
26	GI:349502737 PRODUCT:hypothetical protein	GI:402761529 PRODUCT:hypothetical protein	GI:398256231 PRODUCT:hypothetical protein		
27	GI:349502738 PRODUCT:putative nuclease SbcCD D subunit	GI:402761530 PRODUCT:hypothetical protein	GI:398256232 PRODUCT:putative nuclease SbcCD D subunit	GI:29135001 PRODUCT:ORF065	
28	GI:349502739 PRODUCT:hypothetical protein	GI:402761531 PRODUCT:hypothetical protein	GI:398256233 PRODUCT:hypothetical protein	GI:29135002 PRODUCT:ORF066 **STR.**	
29	GI:349502740 PRODUCT:hypothetical protein	GI:402761532 PRODUCT:hypothetical protein	GI:398256234 PRODUCT:hypothetical protein	GI:29135003 PRODUCT:ORF067	
30	GI:349502741 PRODUCT:hypothetical protein	GI:402761533 PRODUCT:hypothetical protein	GI:398256235 PRODUCT:hypothetical protein	GI:29135004 PRODUCT:ORF068	

31	GI:349502742 PRODUCT:hypothetical protein	GI:402761534 PRODUCT:hypothetical protein	GI:398256236 PRODUCT:hypothetical protein		
32	GI:349502743 PRODUCT:hypothetical protein	GI:402761535 PRODUCT:hypothetical protein	GI:398256237 PRODUCT:hypothetical protein	GI:29135006 PRODUCT:ORF070	
33	GI:349502744 PRODUCT:hypothetical protein	GI:402761536 PRODUCT:hypothetical protein	GI:398256238 PRODUCT:hypothetical protein		PhiKZ 70_2 small frame homologous to 201ϕ2-1p127 (YP_001956851.1) **STR.**
34	GI:349502745 PRODUCT:putative DNA-directed RNA polymerase beta subunit 2	GI:402761537 PRODUCT:hypothetical protein	GI:398256239 PRODUCT:putative DNA-directed RNA polymerase beta subunit 1	GI:29135007 PRODUCT:ORF071	
35	GI:349502746 PRODUCT:putative DNA-directed RNA polymerase beta' subunit	GI:402761539 PRODUCT:hypothetical protein	GI:398256240 PRODUCT:putative DNA-directed RNA polymerase beta' subunit 1	GI:29135010 PRODUCT:ORF074	
36	GI:349502747 PRODUCT:hypothetical protein	GI:402761540 PRODUCT:phage putative ATP-dependent DNA helicase	GI:398256241 PRODUCT:putative helicase	GI:29135011 PRODUCT:ORF075	
37	GI:349502748 PRODUCT:hypothetical protein	GI:402761541 PRODUCT:hypothetical protein	GI:398256242 PRODUCT:hypothetical protein		
38	GI:349502749 PRODUCT:hypothetical protein	GI:402761542 PRODUCT:hypothetical protein	GI:398256243 PRODUCT:hypothetical protein		
39	GI:349502750 PRODUCT:hypothetical protein	GI:402761543 PRODUCT:hypothetical protein	GI:398256244 PRODUCT:hypothetical protein	GI:29135013 PRODUCT:ORF077	

40	GI:349502751 PRODUCT:hypothetical protein	GI:402761544 PRODUCT:hypothetical protein	GI:398256245 PRODUCT:hypothetical protein		
41	GI:349502752 PRODUCT:hypothetical protein	GI:402761545 PRODUCT:hypothetical protein	GI:398256246 PRODUCT:hypothetical protein		
42	GI:349502753 PRODUCT:putative DNA-directed RNA polymerase beta subunit 3	GI:402761546 PRODUCT:phage putative RNA polymerase beta subunit 3	GI:398256247 PRODUCT:putative DNA-directed RNA polymerase beta' subunit 2	GI:29135016 PRODUCT:ORF080 **STR.**	
43	GI:349502754 PRODUCT:hypothetical protein	GI:402761547 PRODUCT:hypothetical protein	GI:398256248 PRODUCT:hypothetical protein		
44	GI:349502755 PRODUCT:hypothetical protein	GI:402761548 PRODUCT:hypothetical protein	GI:398256249 PRODUCT:hypothetical protein	GI:29135018 PRODUCT:ORF082	
45	GI:349502756 PRODUCT:putative virion structural protein 1	GI:402761549 PRODUCT:putative phage virion structural protein	GI:398256250 PRODUCT:putative virion structural protein 1	GI:29135020 PRODUCT:ORF084 **STR.**	
46	GI:349502757 PRODUCT:hypothetical protein	GI:402761551 PRODUCT:hypothetical protein	GI:398256251 PRODUCT:hypothetical protein		
47	GI:349502758 PRODUCT:hypothetical protein	GI:402761552 PRODUCT:hypothetical protein	GI:398256252 PRODUCT:hypothetical protein		
48	GI:349502759 PRODUCT:putative virion structural protein 2	GI:402761553 PRODUCT:phage virion structural protein	GI:398256253 PRODUCT:putative virion structural protein 2	GI:29135023 PRODUCT:ORF087 **STR.**	
49	GI:349502760 PRODUCT:putative virion structural protein	GI:402761554 PRODUCT:phage virion structural protein	GI:398256254 PRODUCT:putative virion structural protein 3	GI:29135024 PRODUCT:ORF088 **STR.**	

	3				
50	GI:349502761 PRODUCT:putative virion structural protein 4	GI:402761555 PRODUCT:phage virion structural protein	GI:398256255 PRODUCT:putative virion structural protein 4	GI:29135025 PRODUCT:ORF089 **STR.**	
51	GI:349502762 PRODUCT:putative virion structural protein 5	GI:402761556 PRODUCT:phage virion structural protein	GI:398256256 PRODUCT:putative virion structural protein 5	GI:29135026 PRODUCT:ORF090 **STR.**	
52	GI:349502763 PRODUCT:hypothetical protein	GI:402761557 PRODUCT:hypothetical protein	GI:398256257 PRODUCT:hypothetical protein		
53	GI:349502764 PRODUCT:hypothetical protein	GI:402761558 PRODUCT:hypothetical protein	GI:398256258 PRODUCT:hypothetical protein		gp54 is paralog of gp53, both gp53 and ϕKZ gp93 are members of pfam12699 **STR.**
54	GI:349502765 PRODUCT:hypothetical protein	GI:402761559 PRODUCT:hypothetical protein	GI:398256259 PRODUCT:hypothetical protein	GI:29134971 PRODUCT:ORF035	pfam12699 **STR.**
58	GI:349502769 PRODUCT:putative dihydrofolate reductase			GI:29134940 PRODUCT:ORF004	
61	GI:349502772 PRODUCT:hypothetical protein	GI:402761566 PRODUCT:hypothetical protein	GI:398256263 PRODUCT:hypothetical protein	GI:29135034 PRODUCT:ORF098 **STR.**	
62	GI:349502773 PRODUCT:putative virion structural protein 6	GI:402761567 PRODUCT:phage virion structural protein	GI:398256264 PRODUCT:putative virion structural protein 6	GI:29135035 PRODUCT:ORF099 **STR.**	

63	GI:349502774 PRODUCT:hypothetical protein	GI:402761568 PRODUCT:hypothetical protein	GI:398256265 PRODUCT:hypothetical protein		
64	GI:349502775 PRODUCT:putative virion structural protein 7	GI:402761569 PRODUCT:hypothetical protein	GI:398256266 PRODUCT:putative virion structural protein 7	GI:29135037 PRODUCT:ORF101 **STR.**	
65	GI:349502776 PRODUCT:hypothetical protein	GI:402761570 PRODUCT:hypothetical protein	GI:398256267 PRODUCT:hypothetical protein		
73	GI:349502784 PRODUCT:putative helicase	GI:402761579 PRODUCT:phage putative helicase	GI:398256273 PRODUCT:putative helicase	GI:29135054 PRODUCT:ORF118	
74	GI:349502785 PRODUCT:hypothetical protein	GI:402761580 PRODUCT:hypothetical protein	GI:398256274 PRODUCT:hypothetical protein		
75	GI:349502786 PRODUCT:putative major capsid protein	GI:402761582 PRODUCT:phage major capsid protein	GI:398256275 PRODUCT:putative major capsid protein	GI:29135056 PRODUCT:ORF120 **STR.**	
76	GI:349502787 PRODUCT:hypothetical protein	GI:402761584 PRODUCT:hypothetical protein	GI:398256276 PRODUCT:hypothetical protein		
77	GI:349502788 PRODUCT:hypothetical protein	GI:402761323 PRODUCT:hypothetical protein	GI:398256277 PRODUCT:hypothetical protein	GI:29135059 PRODUCT:ORF123	
78	GI:349502789 PRODUCT:hypothetical protein	GI:402761324 PRODUCT:hypothetical protein	GI:398256278 PRODUCT:hypothetical protein		
81	GI:349502792 PRODUCT:putative virion structural protein 8	GI:402761326 PRODUCT:hypothetical protein	GI:398256279 PRODUCT:putative virion structural protein 8	GI:29135065 PRODUCT:ORF129 **STR.**	

82	GI:349502793 PRODUCT:putative virion structural protein 9	GI:402761327 PRODUCT:putative virion structural protein	GI:398256280 PRODUCT:putative virion structural protein 9	GI:29135064 PRODUCT:ORF128 **STR.**	
83	GI:349502794 PRODUCT:hypothetical protein	GI:402761328 PRODUCT:hypothetical protein	GI:398256281 PRODUCT:hypothetical protein		GI:29135063 PRODUCT:ORF127 **STR.**
84	GI:349502795 PRODUCT:putative virion structural protein 10	GI:402761329 PRODUCT:putative virion structural protein	GI:398256282 PRODUCT:putative virion structural protein 10	GI:29135075 PRODUCT:ORF139 **STR.**	
85	GI:349502796 PRODUCT:hypothetical protein	GI:402761330 PRODUCT:hypothetical protein	GI:398256283 PRODUCT:hypothetical protein	GI:29135076 PRODUCT:ORF140	
86	GI:349502797 PRODUCT:hypothetical protein	GI:402761331 PRODUCT:hypothetical protein	GI:398256284 PRODUCT:hypothetical protein		
88	GI:349502799 PRODUCT:putative acetyltransferase	GI:402761333 PRODUCT:putative acetyltransferase	GI:398256286 PRODUCT:putative acetyltransferase		
89	GI:349502800 PRODUCT:hypothetical protein	GI:402761334 PRODUCT:hypothetical protein	GI:398256287 PRODUCT:hypothetical protein		
91	GI:349502802 PRODUCT:hypothetical protein	GI:402761336 PRODUCT:hypothetical protein	GI:398256289 PRODUCT:hypothetical protein		
92	GI:349502803 PRODUCT:hypothetical protein	GI:402761337 PRODUCT:hypothetical protein	GI:398256290 PRODUCT:hypothetical protein		GI:29135143 PRODUCT:ORF207
93	GI:349502804 PRODUCT:hypothetical protein	GI:402761338 PRODUCT:hypothetical protein	GI:398256292 PRODUCT:hypothetical protein		

94	GI:349502805 PRODUCT:hypothetical protein	GI:402761339 PRODUCT:hypothetical protein	GI:398256297 PRODUCT:hypothetical protein		
95	GI:349502806 PRODUCT:putative GNAT family acetyltransferase	GI:402761341 PRODUCT:putative GNAT family acetyltransferase	GI:398256298 PRODUCT:putative ribosomal-protein-alanine N-acetyltransferase		
98	GI:349502809 PRODUCT:hypothetical protein	GI:402761343 PRODUCT:hypothetical protein	GI:398256301 PRODUCT:hypothetical protein		
100	GI:349502811 PRODUCT:putative thymidylate kinase	GI:402761346 PRODUCT:putative thymidylate kinase	GI:398256302 PRODUCT:putative thymidylate kinase		
108	GI:349502819 PRODUCT:hypothetical protein	GI:402761352 PRODUCT:hypothetical protein	GI:398256306 PRODUCT:hypothetical protein		
109	GI:349502820 PRODUCT:hypothetical protein	GI:402761353 PRODUCT:hypothetical protein	GI:398256323 PRODUCT:hypothetical protein		
115	GI:349502826 PRODUCT:hypothetical protein	GI:402761359 PRODUCT:hypothetical protein	GI:398256327 PRODUCT:hypothetical protein		
118	GI:349502829 PRODUCT:hypothetical protein	GI:402761362 PRODUCT:hypothetical protein	GI:398256329 PRODUCT:hypothetical protein		
120	GI:349502831 PRODUCT:hypothetical protein	GI:402761364 PRODUCT:hypothetical protein	GI:398256333 PRODUCT:hypothetical protein	GI:29135087 PRODUCT:ORF151	
127	GI:349502838 PRODUCT:hypothetical protein	GI:402761367 PRODUCT:hypothetical protein	GI:398256334 PRODUCT:hypothetical protein		
138	GI:349502849 PRODUCT:hypothetical protein	GI:402761380 PRODUCT:putative virion structural protein	GI:398256338 PRODUCT:hypothetical protein	GI:29135062 PRODUCT:ORF126 **STR.**	

139	GI:349502850 PRODUCT:hypothetical protein	GI:402761381 PRODUCT:putative virion structural protein	GI:398256339 PRODUCT:hypothetical protein		GI:29135063 PRODUCT:ORF127 **STR.**
140	GI:349502851 PRODUCT:putative virion structural protein 11	GI:402761382 PRODUCT:putative virion structural protein	GI:398256340 PRODUCT:putative virion structural protein 11	GI:29135063 PRODUCT:ORF127 **STR.**	
141	GI:349502852 PRODUCT:hypothetical protein	GI:402761383 PRODUCT:putative virion structural protein	GI:398256341 PRODUCT:hypothetical protein	GI:29135031 PRODUCT:ORF095 **STR.**	
142	GI:349502853 PRODUCT:hypothetical protein	GI:402761384 PRODUCT:hypothetical protein	GI:398256344 PRODUCT:hypothetical protein		GI:29135063 PRODUCT:ORF127 **STR.**
143	GI:349502854 PRODUCT:hypothetical protein	GI:402761385 PRODUCT:putative virion structural protein	GI:398256343 PRODUCT:hypothetical protein		GI:29135063 PRODUCT:ORF127 **STR.**
144	GI:349502855 PRODUCT:hypothetical protein	GI:402761386 PRODUCT:hypothetical protein	GI:398256346 PRODUCT:hypothetical protein		GI:29135063 PRODUCT:ORF127 **STR.**
145	GI:349502856 PRODUCT:putative virion structural protein 12			GI:29135019 PRODUCT:ORF083 **STR.**	
146	GI:349502857 PRODUCT:putative virion structural protein 13	GI:402761388 PRODUCT:putative virion structural protein	GI:398256350 PRODUCT:putative virion structural protein 13		GI:29135019 PRODUCT:ORF083 **STR.**
147	GI:349502858 PRODUCT:putative virion structural protein 14				GI:29135019 PRODUCT:ORF083 **STR.**

148	GI:349502859 PRODUCT:putative virion structural protein 15	GI:402761393 PRODUCT:putative virion structural protein	GI:398256349 PRODUCT:putative virion structural protein 12		GI:29135019 PRODUCT:ORF083 **STR.**
149	GI:349502860 PRODUCT:hypothetical protein				GI:29135019 PRODUCT:ORF083 **STR.**
150	GI:349502861 PRODUCT:putative virion structural protein 16	GI:402761392 PRODUCT:putative virion structural protein	GI:398256351 PRODUCT:putative virion structural protein 14		GI:29135019 PRODUCT:ORF083 **STR.**
151	GI:349502862 PRODUCT:putative virion structural protein 17				GI:29135019 PRODUCT:ORF083 **STR.**
152	GI:349502863 PRODUCT:putative virion structural protein 18				GI:29135019 PRODUCT:ORF083 **STR.**
153	GI:349502863 PRODUCT:putative virion structural protein 19				GI:29135019 PRODUCT:ORF083 **STR.**
154	GI:349502864 PRODUCT:hypothetical protein				GI:29135063 PRODUCT:ORF127 **STR.**
157	GI:349502868 PRODUCT:hypothetical protein	GI:402761398 PRODUCT:phage tail assembly-like protein	GI:398256354 PRODUCT:hypothetical protein		
160	GI:349502871 PRODUCT:hypothetical protein	GI:402761400 PRODUCT:hypothetical protein	GI:398256352 PRODUCT:hypothetical protein		

161	GI:349502872 PRODUCT:hypothetical protein	GI:402761402 PRODUCT:hypothetical protein	GI:398256355 PRODUCT:hypothetical protein		
162	GI:349502873 PRODUCT:hypothetical protein	GI:402761403 PRODUCT:hypothetical protein	GI:398256356 PRODUCT:hypothetical protein		
163	GI:349502874 PRODUCT:putative radical SAM superfamily protein 1	GI:402761404 PRODUCT:putative radical SAM superfamily protein	GI:398256357 PRODUCT:putative radical SAM superfamily protein 1		
164	GI:349502875 PRODUCT:putative radical SAM superfamily protein 2	GI:402761405 PRODUCT:putative radical SAM superfamily protein 2	GI:398256358 PRODUCT:putative radical SAM superfamily protein 2		
165	GI:349502876 PRODUCT:hypothetical protein	GI:402761406 PRODUCT:hypothetical protein	GI:398256359 PRODUCT:hypothetical protein		
167	GI:349502878 PRODUCT:putative virion structural protein 20	GI:402761410 PRODUCT:putative virion structural protein	GI:398256362 PRODUCT:putative virion structural protein 15	GI:29135070 PRODUCT:ORF134 **STR.**	
168	GI:349502879 PRODUCT:hypothetical protein	GI:402761411 PRODUCT:hypothetical protein	GI:398256363 PRODUCT:hypothetical protein	GI:29135081 PRODUCT:ORF145 **STR.**	
169	GI:349502880 PRODUCT:hypothetical protein	GI:402761412 PRODUCT:hypothetical protein	GI:398256364 PRODUCT:hypothetical protein	GI:29135069 PRODUCT:ORF133 **STR.**	
170	GI:349502881 PRODUCT:hypothetical protein	GI:402761413 PRODUCT:hypothetical protein	GI:398256365 PRODUCT:hypothetical protein	GI:29135067 PRODUCT:ORF131 **STR.**	
171	GI:349502882 PRODUCT:hypothetical protein	GI:402761414 PRODUCT:hypothetical protein	GI:398256366 PRODUCT:hypothetical protein	GI:29135066 PRODUCT:ORF130 **STR.**	

172	GI:349502883 PRODUCT:hypothetical protein	GI:402761415 PRODUCT:hypothetical protein	GI:398256367 PRODUCT:hypothetical protein		
173	GI:349502884 PRODUCT:putative structural protein	GI:402761416 PRODUCT:putative structural protein	GI:398256368 PRODUCT:putative structural protein	GI:29135100 PRODUCT:ORF164 **STR.**	
175	GI:349502886 PRODUCT:hypothetical protein	GI:402761418 PRODUCT:hypothetical protein	GI:398256369 PRODUCT:hypothetical protein		
183	GI:349502894 PRODUCT:hypothetical protein	GI:402761428 PRODUCT:hypothetical protein	GI:398256409 PRODUCT:hypothetical protein		
186	GI:349502897 PRODUCT:putative SMC domain-containing protein	GI:402761431 PRODUCT:putative SMC domain-containing protein	GI:398256374 PRODUCT:putative SMC domain-containing protein	GI:29135101 PRODUCT:ORF165	
196	GI:349502907 PRODUCT:hypothetical protein	GI:402761440 PRODUCT:hypothetical protein	GI:398256381 PRODUCT:hypothetical protein	GI:29135040 PRODUCT:ORF104	
198	GI:349502909 PRODUCT:putative endolysin	GI:402761441 PRODUCT:putative endolysin	GI:398256382 PRODUCT:putative endolysin		GI:29135080 PRODUCT: endolysin
199	GI:349502910 PRODUCT:hypothetical protein	GI:402761442 PRODUCT:hypothetical protein	GI:398256383 PRODUCT:hypothetical protein		
202	GI:349502913 PRODUCT:hypothetical protein	GI:402761444 PRODUCT:hypothetical protein	GI:398256385 PRODUCT:hypothetical protein	GI:29135097 PRODUCT:ORF161 **STR.**	
203	GI:349502914 PRODUCT:putative virion structural protein 21	GI:402761445 PRODUCT:putative virion structural protein	GI:398256386 PRODUCT:putative virion structural protein 16	GI:29135093 PRODUCT:ORF157 **STR.**	

204	GI:349502915 PRODUCT:hypothetical protein	GI:402761446 PRODUCT:hypothetical protein	GI:398256387 PRODUCT:hypothetical protein		
205	GI:349502916 PRODUCT:hypothetical protein	GI:402761447 PRODUCT:hypothetical protein	GI:398256388 PRODUCT:hypothetical protein		GI:29135092 PRODUCT:ORF156
206	GI:349502917 PRODUCT:putative endodeoxyribonuclease	GI:402761448 PRODUCT:putative endodeoxyribonuclease	GI:398256389 PRODUCT:putative endodeoxyribonuclease		
207	GI:349502918 PRODUCT:putative ribonuclease H	GI:402761449 PRODUCT:putative ribonuclease H	GI:398256390 PRODUCT:putative ribonuclease H	GI:29135091 PRODUCT:ORF155	
209	GI:349502920 PRODUCT:hypothetical protein	GI:402761451 PRODUCT:hypothetical protein	GI:398256391 PRODUCT:hypothetical protein		
212	GI:349502923 PRODUCT:hypothetical protein	GI:402761455 PRODUCT:hypothetical protein	GI:398256393 PRODUCT:hypothetical protein		
214	GI:349502925 PRODUCT:hypothetical protein	GI:402761458 PRODUCT:hypothetical protein	GI:398256394 PRODUCT:hypothetical protein	GI:29135089 PRODUCT:ORF153 **STR.**	
216	GI:349502927 PRODUCT:hypothetical protein	GI:402761460 PRODUCT:hypothetical protein	GI:398256395 PRODUCT:hypothetical protein	GI:29135088 PRODUCT:ORF152	
218	GI:349502929 PRODUCT:putative virion structural protein 22	GI:402761461 PRODUCT:hypothetical protein	GI:398256397 PRODUCT:putative virion structural protein 17	GI:29135085 PRODUCT:ORF149 **STR.**	
219	GI:349502930 PRODUCT:hypothetical protein	GI:402761344 PRODUCT:hypothetical protein	GI:398256398 PRODUCT:hypothetical protein	GI:29135083 PRODUCT:ORF147	
220	GI:349502931 PRODUCT:hypothetical protein			GI:29135105 PRODUCT:ORF169	

221	GI:349502932 PRODUCT:hypothetical protein	GI:402761463 PRODUCT:hypothetical protein	GI:398256400 PRODUCT:hypothetical protein		
222	GI:349502933 PRODUCT:hypothetical protein	GI:402761464 PRODUCT:hypothetical protein	GI:398256401 PRODUCT:hypothetical protein		
223	GI:349502934 PRODUCT:hypothetical protein	GI:402761465 PRODUCT:hypothetical protein	GI:398256402 PRODUCT:hypothetical protein	GI:29135110 PRODUCT:ORF174 **STR.**	
224	GI:349502935 PRODUCT:hypothetical protein	GI:402761466 PRODUCT:hypothetical protein	GI:398256403 PRODUCT:hypothetical protein		
225	GI:349502936 PRODUCT:hypothetical protein	GI:402761467 PRODUCT:hypothetical protein	GI:398256404 PRODUCT:hypothetical protein		
229	GI:349502940 PRODUCT:thymidylate synthase	GI:402761471 PRODUCT:thymidylate synthase	GI:398256311 PRODUCT:thymidylate synthase	GI:29135171 PRODUCT:ORF235	
233	GI:349502944 PRODUCT:hypothetical protein	GI:402761473 PRODUCT:hypothetical protein	GI:398256319 PRODUCT:hypothetical protein	GI:29135233 PRODUCT:ORF297	
234	GI:349502945 PRODUCT:hypothetical protein	GI:402761474 PRODUCT:hypothetical protein	GI:398256317 PRODUCT:hypothetical protein		
235	GI:349502946 PRODUCT:hypothetical protein	GI:402761475 PRODUCT:hypothetical protein	GI:398256320 PRODUCT:hypothetical protein		
237	GI:349502948 PRODUCT:hypothetical protein				GI:29135063 PRODUCT:ORF127 **STR.**
238	GI:349502949 PRODUCT:hypothetical protein	GI:402761479 PRODUCT:hypothetical protein	GI:398256410 PRODUCT:hypothetical protein	GI:29135118 PRODUCT:ORF182 **STR.**	

239	GI:349502950 PRODUCT:putative tail fibre protein	GI:402761480 PRODUCT:putative phage tail fiber protein	GI:398256411 PRODUCT:putative tail fiber protein	GI:29135080 PRODUCT:ORF144 **STR.**	
240	GI:349502951 PRODUCT:putative DNA-directed RNA polymerase beta subunit 4	GI:402761481 PRODUCT:putative phage DNA-directed RNA polymerase beta subunit	GI:398256412 PRODUCT:putative DNA-directed RNA polymerase beta subunit 2	GI:29135116 PRODUCT:ORF180 **STR.**	
241	GI:349502952 PRODUCT:putative DNA-directed RNA polymerase beta subunit 5	GI:402761482 PRODUCT:putative phage DNA-directed RNA polymerase beta subunit 2	GI:398256413 PRODUCT:putative DNA-directed RNA polymerase beta subunit 3	GI:29135114 PRODUCT:ORF178 **STR.**	
242	GI:349502953 PRODUCT:hypothetical protein	GI:402761483 PRODUCT:hypothetical protein	GI:398256414 PRODUCT:hypothetical protein		
243	GI:349502954 PRODUCT:hypothetical protein	GI:402761484 PRODUCT:hypothetical protein	GI:398256415 PRODUCT:hypothetical protein	GI:29135113 PRODUCT:ORF177 **STR.**	
244	GI:349502955 PRODUCT:hypothetical protein	GI:402761485 PRODUCT:hypothetical protein	GI:398256416 PRODUCT:hypothetical protein	GI:29135112 PRODUCT:ORF176 **STR.**	
245	GI:349502956 PRODUCT:putative virion structural protein 23	GI:402761486 PRODUCT:putative virion structural protein	GI:398256417 PRODUCT:putative virion structural protein 18	GI:29135111 PRODUCT:ORF175 **STR.**	
246	GI:349502957 PRODUCT:hypothetical protein	GI:402761487 PRODUCT:hypothetical protein	GI:398256418 PRODUCT:hypothetical protein		
247	GI:349502958 PRODUCT:hypothetical protein	GI:402761488 PRODUCT:hypothetical protein	GI:398256419 PRODUCT:hypothetical protein		

248	GI:349502959 PRODUCT:hypothetical protein	GI:402761489 PRODUCT:hypothetical protein	GI:398256420 PRODUCT:hypothetical protein		
251	GI:349502962 PRODUCT:putative HD domain protein	GI:402761491 PRODUCT:putative phage HD domain protein	GI:398256421 PRODUCT:putative HD domain protein		
252	GI:349502963 PRODUCT:hypothetical protein	GI:402761492 PRODUCT:hypothetical protein	GI:398256422 PRODUCT:hypothetical protein		
255	GI:349502966 PRODUCT:putative virion structural protein 24	GI:402761494 PRODUCT:putative virion structural protein	GI:398256423 PRODUCT:putative virion structural protein 19	GI:29134966 PRODUCT:ORF030 **STR.**	
256	GI:349502967 PRODUCT:putative tail sheath protein	GI:402761495 PRODUCT:putative phage tail sheath protein	GI:398256424 PRODUCT:putative tail sheath protein	GI:29134965 PRODUCT:ORF029 **STR.**	
257	GI:349502968 PRODUCT:hypothetical protein	GI:402761496 PRODUCT:hypothetical protein	GI:398256425 PRODUCT:hypothetical protein	GI:29134964 PRODUCT:ORF028	
258	GI:349502969 PRODUCT:putative virion structural protein 25	GI:402761497 PRODUCT:putative virion structural protein	GI:398256426 PRODUCT:putative virion structural protein 20	GI:29134963 PRODUCT:ORF027 **STR.**	
259	GI:349502970 PRODUCT:putative virion structural protein 26	GI:402761498 PRODUCT:putative virion structural protein	GI:398256427 PRODUCT:putative virion structural protein 21	GI:29134962 PRODUCT:ORF026 **STR.**	
260	GI:349502971 PRODUCT:putative terminase large subunit	GI:402761499 PRODUCT:terminase large subunit	GI:398256428 PRODUCT:putative terminase large subunit	GI:29134961 PRODUCT:ORF025	
262	GI:349502973 PRODUCT:hypothetical protein	GI:402761500 PRODUCT:hypothetical protein	GI:398256433 PRODUCT:hypothetical protein	GI:29134968 PRODUCT:ORF032 **STR.**	

263	GI:349502974 PRODUCT:hypothetical protein	GI:402761501 PRODUCT:hypothetical protein	GI:398256434 PRODUCT:hypothetical protein		

Table S2. **Titers and reversion rates of SPN3US amber mutant candidates.** Suppressor strains tested were TT6675 (supD) and TT9079 transformed with pMN suppressor plasmids obtained from Dr. David Peabody, supS, supG and supA refer to serine, glycine and alanine suppressors respectively. Non-suppressor strains used for determination of reversion rate were UB0015 and TT9079.

	**Titer (pfu/ml)**				**Reversion rate**	
**Amber No**	**TT6675 (sup+)**	**TT9079/ pMNsupS**	**TT9079/ pMNsupG**	**TT9079/ pMNsupA**	**UB0015**	**TT9079**
1	3.6E+12	2.4E+12	2.0E+11	No growth	<E-5	<E-5
2	3.8E+12	4.6E+12	6.0E+11	4.0E+11	<E-5	<E-6
3	8.0E+11	5.4E+11	6.0E+10	No growth	<E-6	<E-7
4	3.0E+11	3.2E+11	3.0E+10	No growth	<E-7	E-7
5	2.0E+11	1.6E+11	2.0E+10	No growth	<E-7	<E-7
6	8.0E+11	2.4E+11	8.0E+10	3.2E+10	<E-6	E-6
7	5.0E+11	2.4E+11	2.0E+10	No growth	E-7	E-5
8	2.0E+11	6.0E+10	1.0E+10	No growth	<E-6	<E-6
9	3.6E+11	2.4E+11	2.0E+10	No growth	<E-6	<E-6
10	1.0E+11	4.8E+11	1.0E+11	1.2E+11	<E-6	<E-6
11	2.6E+10	2.2E+11	1.0E+11	2.2E+10	<E-5	<E-5
12	2.2E+11	2.4E+11	6.0E+10	6.0E+10	<E-6	<E-6
13	2.4E+12	? E+11	1.0E+11	turbid?	<E-6	<E-6
14	2.0E+11	1.0E+11	4.0E+11	1.6E+11	<E-6	<E-7
15	2.0E+11	2.0E+11	2.0E+10	No growth	<E-7	E-7
16	No growth	No growth	No growth	No growth		
17	2.2E+11	1.6E+11	4.0E+10	No growth	<E-5	E-6
18	1.6E+12	7.0E+11	No growth	No growth	<E-5	E-7
19	1.0E+12	1.0E+12	1.4E+11	No growth	<E-5	E-6
20	1.0E+12	5.2E+11	2.0E+11	1.8E+11	E-6	<E-6
21	1.8E+12	2.0E+11	4.0E+11	2.0E+11	<E-6	<E-6
22	1.8E+12	1.6E+12	2.0E+11	No growth	<E-7	<E-7
23	1.8E+12	4.0E+12	8.0E+11	No growth	<E-7	<E-7
24	1.0E+12	8.0E+11	4.0E+11	No growth	<E-7	<E-7
25	1.0E+12	1.0E+12	2.0E+11	1.6E+11	<E-6	<E-6
26	1.0E+12	8.0E+11	2.6E+11	No growth	E-7	E-7
27	8.4E+11	5.6E+11	1.6E+11	4.0E+10	<E-6	E-4
28	1.6E+11	5.4E+11	3.0E+11	No growth	E-4	E-4

29	2.0E+11	9.0E+11	3.0E+11	No growth	<E-5	<E-5
30	1.2E+11	6.0E+11	3.0E+11	2.6E+11	<E-4	E-6
31	2.2E+11	8.4E+11	3.6E+11	1.6E+11	<E-4	<E-5
32	1.4E+11	5.8E+11	2.0E+11	No growth	<E-4	<E-5
33	1.2E+11	5.4E+11	3.0E+11	Turbid zone	<E-5	<E-5
34	1.0E+11	5.2E+11	1.0E+11	No growth	<E-5	<E-5
35	8.2E+11	4.8E+11	1.2E+11	Turbid zone	<E-5	<E-5
36	1.2E+11	6.2E+11	4.2E+11	No growth	<E-5	E-6
37	1.2E+12	4.0E+11	6.0E+11	1.0E+11	1.7E-06	1.7E-06
38	1.2E+12	8.0E+11	3.6E+11	1.0E+11	1.7E-05	1.7E-05
39	2.0E+12	2.2E+12	2.0E+12	No growth	1.0E-05	1.0E-05
40	1.4E+12	1.8E+12	6.0E+11	No growth	1.4E-05	E-5
41	1.4E+12	1.8E+12	6.0E+11	6.0E+10	1.4E-05	1.4E-05
42	3.6E+12	1.2E+12	2.0E+11	4.0E+11	1.6E-05	1.6E-05
43	1.6E+12	8.0E+11	2.6E+12	1.2E+12	1.3E-05	1.3E-06
44	4.4E+12	2.0E+12	4.0E+11	4.0E+11	5.0E-07	5.0E-07
45	3.6E+12	1.6E+12	1.4E+12	2.0E+10	1.6E-05	1.6E-05
46	4.6E+12	1.0E+12	8.0E+11	No growth	4.3E-06	4.3E-06
47	3.6E+12	4.0E+12	1.6E+12	2.0E+11	<E-6	<E-7
48	9.6E+12	2.6E+12	1.2E+12	No growth	<E-6	<E-7
49	2.0E+11	4.0E+11	1.0E+11	2.0E+11	<E-6	<E-6
50	4.6E+12	4.0E+11	No growth	1.0E+11	<E-7	<E-7
51	8.0E+11	8.0E+11	8.0E+10	4.0E+11	<E-5	

Table S3. Mutations identified in SPN3US amber mutants.

**am1**	**Reference Position**	**Type**	**Reference Base**	**Called Base**	**Impact**	**SNP %**	**P not ref**	**Q call**	**Feature Type**	**Feature Name**	**DNA Change**	**Amino Acid Change**
	46330	SNP	C	T	Nonsense	99.80%	100.00%	60	CDS	SPN3US_0047	c.1432C>T	p.Q478.
	56866	SNP	C	T	Synonymous	99.70%	100.00%	60	CDS	SPN3US_0055	c.504C>T	p.T168T
	142448	SNP	C	T	Non Synonymous	99.40%	100.00%	60	CDS	SPN3US_0165	c.721G>A	p.A241T
	187963	SNP	G	A	Non Synonymous	100.00%	100.00%	60	CDS	SPN3US_0216	c.13G>A	p.A5T
	189706	SNP	C	T	Synonymous	99.80%	100.00%	60	CDS	SPN3US_0217	c.216C>T	p.Y72Y
	201785	SNP	G	A	Non Synonymous	99.60%	100.00%	60	CDS	SPN3US_0235	c.815G>A	p.G272D
	239940	Ins	-	T		91.90%	100.00%	60				
**am2**	**Reference Position**	**Type**	**Reference Base**	**Called Base**	**Impact**	**SNP %**	**P not ref**	**Q call**	**Feature Type**	**Feature Name**	**DNA Change**	**Amino Acid Change**
	320	SNP	C	T		99.50%	100.00%	60				
	63849	SNP	C	T	Nonsense	99.60%	100.00%	60	CDS	SPN3US_0064	c.763C>T	p.Q255.
	77968	Ins	-	T		85.30%	100.00%	60				
	93454	Ins	-	T		89.60%	100.00%	60				
	98358	SNP	C	T	Non Synonymous	99.90%	100.00%	60	CDS	SPN3US_0106	c.680C>T	p.A227V
	103056	SNP	G	A	Non Synonymous	100.00%	100.00%	60	CDS	SPN3US_0116	c.173G>A	p.R58H
	122733	SNP	G	A	Non Synonymous	99.90%	100.00%	60	CDS	SPN3US_0145	c.1216G>A	p.V406M
	239933	Ins	-	T		82.90%	100.00%	60				
**am3**	**Reference Position**	**Type**	**Reference Base**	**Called Base**	**Impact**	**SNP %**	**P not ref**	**Q call**	**Feature Type**	**Feature Name**	**DNA Change**	**Amino Acid**

												**Change**
	77022	SNP	C	T	Nonsense	99.60%	100.00%	60	CDS	SPN3US_0078	c.1459C>T	p.Q487.
	89936	SNP	C	T	Non Synonymous	99.80%	100.00%	60	CDS	SPN3US_0094	c.575C>T	p.T192I
	91091	SNP	C	T	Non Synonymous	99.60%	100.00%	60	CDS	SPN3US_0096	c.131C>T	p.P44L
	131809	SNP	C	T		99.50%	100.00%	60				
	217260	SNP	G	A	Non Synonymous	99.80%	100.00%	60	CDS	SPN3US_0241	c.3272G>A	p.G1091D
	239933	Ins	-	T		98.70%	100.00%	60				
**am6**	**Reference Position**	**Type**	**Reference Base**	**Called Base**	**Impact**	**SNP %**	**P not ref**	**Q call**	**Feature Type**	**Feature Name**	**DNA Change**	**Amino Acid Change**
	1398	SNP	C	T	Synonymous	99.50%	100.00%	60	CDS	SPN3US_0001	c.213C>T	p.G71G
	20159	SNP	C	T	Nonsense	99.50%	100.00%	60	CDS	SPN3US_0025	c.184C>T	p.Q62.
	29178	SNP	C	T	Synonymous	99.80%	100.00%	60	CDS	SPN3US_0034	c.165C>T	p.Y55Y
	42136	SNP	C	T	Synonymous	99.60%	100.00%	60	CDS	SPN3US_0044	c.873G>A	p.P291P
	45934	SNP	G	A	Non Synonymous	99.10%	100.00%	60	CDS	SPN3US_0047	c.1036G>A	p.D346N
	66329	SNP	C	T	Non Synonymous	99.80%	100.00%	60	CDS	SPN3US_0069	c.272C>T	p.P91L
	76751	SNP	G	C	Synonymous	99.80%	100.00%	60	CDS	SPN3US_0078	c.1188G>C	p.A396A
	77948	SNP	C	T		99.70%	100.00%	60				
	85090	SNP	C	T	Non Synonymous	99.60%	100.00%	60	CDS	SPN3US_0086	c.113C>T	p.T38I
	118020	SNP	A	G	Non Synonymous	99.60%	100.00%	60	CDS	SPN3US_0141	c.100A>G	p.T34A
	120459	SNP	C	T	Synonymous	99.70%	100.00%	60	CDS	SPN3US_0143	c.708C>T	p.S236S
	176950	SNP	C	T	Non Synonymous	99.80%	100.00%	60	CDS	SPN3US_0201	c.202C>T	p.R68C
	179383	SNP	C	T	Non Synonymous	99.00%	100.00%	60	CDS	SPN3US_0203	c.571G>A	p.G191R

	215102	SNP	G	A	Non Synonymous	99.80%	100.00%	60	CDS	SPN3US_0241	c.1114G>A	p.A372T
	217208	SNP	G	A	Non Synonymous	99.30%	100.00%	60	CDS	SPN3US_0241	c.3220G>A	p.D1074N
	234789	SNP	G	A	Non Synonymous	99.70%	100.00%	60	CDS	SPN3US_0260	c.427G>A	p.E143K
	240249	SNP	G	A		99.40%	100.00%	60				
**am11**	**Reference Position**	**Type**	**Reference Base**	**Called Base**	**Impact**	**SNP %**	**P not ref**	**Q call**	**Feature Type**	**Feature Name**	**DNA Change**	**Amino Acid Change**
	97805	SNP	G	A	Non Synonymous	99.60%	100.00%	60	CDS	SPN3US_0106	c.127G>A	p.D43N
	111698	SNP	C	T	Non Synonymous	99.90%	100.00%	60	CDS	SPN3US_0129	c.191C>T	p.S64L
	159949	SNP	C	T	Non Synonymous	99.20%	100.00%	60	CDS	SPN3US_0171	c.1211C>T	p.A404V
	167489	SNP	G	A	Synonymous	100.00%	100.00%	60	CDS	SPN3US_0183	c.381G>A	p.Q127Q
	215999	SNP	C	T	Nonsense	100.00%	100.00%	60	CDS	SPN3US_0241	c.2011C>T	p.Q671.
	223314	SNP	C	T		100.00%	100.00%	60				
	225192	SNP	G	A	Non Synonymous	99.50%	100.00%	60	CDS	SPN3US_0252	c.269G>A	p.S90N
	239933	Ins	-	T		97.50%	100.00%	60				
**am13**	**Reference Position**	**Type**	**Reference Base**	**Called Base**	**Impact**	**SNP %**	**P not ref**	**Q call**	**Feature Type**	**Feature Name**	**DNA Change**	**Amino Acid Change**
	66658	SNP	C	T	Nonsense	99.80%	100.00%	60	CDS	SPN3US_0070	c.181C>T	p.Q61.
	87716	Ins	-	C		74.70%	100.00%	60				
	98642	SNP	G	A	Non Synonymous	100.00%	100.00%	60	CDS	SPN3US_0107	c.151G>A	p.D51N
	125063	SNP	A	T	Synonymous	100.00%	100.00%	60	CDS	SPN3US_0148	c.177A>T	p.G59G
	125066	SNP	T	C	Synonymous	100.00%	100.00%	60	CDS	SPN3US_0148	c.180T>C	p.F60F
	125067	SNP	G	T	Non	99.60%	100.00%	60	CDS	SPN3US_0148	c.181G>T	p.A61S

					Synonymous							
	125075	SNP	A	G	Synonymous	100.00%	100.00%	60	CDS	SPN3US_0148	c.189A>G	p.E63E
	125076	SNP	A	T	Non Synonymous	100.00%	100.00%	60	CDS	SPN3US_0148	c.190A>T	p.I64L
	125077	SNP	T	G	Non Synonymous	100.00%	100.00%	60	CDS	SPN3US_0148	c.191T>G	p.I64R
	125078	*SNP*	*A*	*C*	*Synonymous*	*100.00%*	*100.00%*	*60*	*CDS*	*SPN3US_0148*	*c.192A>C*	*p.I64I*
	125084	*SNP*	*A*	*T*	*Synonymous*	*100.00%*	*100.00%*	*60*	*CDS*	*SPN3US_0148*	*c.198A>T*	*p.V66V*
	156655	SNP	C	T	Non Synonymous	100.00%	100.00%	60	CDS	SPN3US_0170	c.2017G>A	p.V673I
	163552	SNP	C	T	Synonymous	99.60%	100.00%	60	CDS	SPN3US_0176	c.223C>T	p.L75L
	208784	SNP	G	A	Non Synonymous	99.80%	100.00%	60	CDS	SPN3US_0239	c.3544C>T	p.L1182F
	239933	Ins	-	T		98.30%	100.00%	60				
**am18**	**Reference Position**	**Type**	**Reference Base**	**Called Base**	**Impact**	**SNP %**	**P not ref**	**Q call**	**Feature Type**	**Feature Name**	**DNA Change**	**Amino Acid Change**
	53558	SNP	C	T		99.60%	100.00%	60				
	109538	SNP	G	A	Non Synonymous	99.60%	100.00%	60	CDS	SPN3US_0124	c.2713G>A	p.V905M
	134633	SNP	G	A	Non Synonymous	99.50%	100.00%	60	CDS	SPN3US_0155	c.1465G>A	p.G489S
	150861	SNP	G	A	Non Synonymous	99.60%	100.00%	60	CDS	SPN3US_0169	c.4085C>T	p.T1362I
	161758	SNP	C	T	Synonymous	100.00%	100.00%	60	CDS	SPN3US_0174	c.166C>T	p.L56L
	179577	SNP	C	T	Nonsense	99.80%	100.00%	60	CDS	SPN3US_0203	c.377G>A	p.W126.
	197542	SNP	G	A	Non Synonymous	99.60%	100.00%	60	CDS	SPN3US_0228	c.803G>A	p.G268D
	219687	SNP	G	A	Non Synonymous	99.80%	100.00%	60	CDS	SPN3US_0243	c.1156G>A	p.A386T
	225627	SNP	G	A		99.90%	100.00%	60				
	239933	Ins	-	T		98.40%	100.00%	60				

-	-	-	-	-	-	-	-	-	-	-	-	-
**am19**	**Reference Position**	**Type**	**Reference Base**	**Called Base**	**Impact**	**SNP %**	**P not ref**	**Q call**	**Feature Type**	**Feature Name**	**DNA Change**	**Amino Acid Change**
	48821	SNP	G	A	Non Synonymous	99.50%	100.00%	60	CDS	SPN3US_0048	c.802C>T	p.P268S
	93455	Ins	-	T		89.90%	100.00%	60				
	178201	SNP	G	A	Synonymous	99.90%	100.00%	60	CDS	SPN3US_0202	c.363C>T	p.V121V
	179287	SNP	G	A	Nonsense	99.80%	100.00%	60	CDS	SPN3US_0203	c.667C>T	p.Q223.
	179288	SNP	G	A	Synonymous	99.80%	100.00%	60	CDS	SPN3US_0203	c.666C>T	p.D222D
	189407	SNP	A	G	Non Synonymous	99.90%	100.00%	60	CDS	SPN3US_0216	c.1457A>G	p.D486G
	239301	SNP	G	A	Non Synonymous	99.60%	100.00%	60	CDS	SPN3US_0263	c.1073G>A	p.S358N
	239933	Ins	-	T		96.50%	100.00%	60				
	240020	SNP	C	T		99.70%	100.00%	60				
**am22**	**Reference Position**	**Type**	**Reference Base**	**Called Base**	**Impact**	**SNP %**	**P not ref**	**Q call**	**Feature Type**	**Feature Name**	**DNA Change**	**Amino Acid Change**
	23427	SNP	C	T	Nonsense	100.00%	100.00%	60	CDS	SPN3US_0029	c.358C>T	p.Q120.
	191277	SNP	G	A	Non Synonymous	99.80%	100.00%	60	CDS	SPN3US_0220	c.298C>T	p.H100Y
	239933	Ins	-	T		63.50%	90.00%	41.7				
**am27**	**Reference Position**	**Type**	**Reference Base**	**Called Base**	**Impact**	**SNP %**	**P not ref**	**Q call**	**Feature Type**	**Feature Name**	**DNA Change**	**Amino Acid Change**
	1658	SNP	C	T	Non Synonymous	99.50%	100.00%	60	CDS	SPN3US_0001	c.473C>T	p.T158I
	63957	SNP	C	T	Nonsense	99.50%	100.00%	60	CDS	SPN3US_0064	c.871C>T	p.Q291.
	75215	SNP	G	A	Non Synonymous	99.50%	100.00%	60	CDS	SPN3US_0077	c.1462G>A	p.G488S
	88765	SNP	C	T	Non	99.50%	100.00%	60	CDS	SPN3US_0092	c.317C>T	p.P106L

					Synonymous							
	100963	SNP	C	T	Nonsense	99.90%	100.00%	60	CDS	SPN3US_0112	c.100C>T	p.Q34.
	133692	SNP	C	T	Non Synonymous	99.90%	100.00%	60	CDS	SPN3US_0155	c.524C>T	p.A175V
	171967	SNP	C	T	Synonymous	99.60%	100.00%	60	CDS	SPN3US_0189	c.102C>T	p.I34I
	206491	SNP	G	A	Non Synonymous	99.60%	100.00%	60	CDS	SPN3US_0239	c.5837C>T	p.S1946L
	239933	Ins	-	T		96.50%	100.00%	60				
**am39**	**Reference Position**	**Type**	**Reference Base**	**Called Base**	**Impact**	**SNP %**	**P not ref**	**Q call**	**Feature Type**	**Feature Name**	**DNA Change**	**Amino Acid Change**
	169242	SNP	C	T	Nonsense	99.50%	100.00%	60	CDS	SPN3US_0186	c.343C>T	p.Q115.
	198993	SNP	C	T	Non Synonymous	99.80%	100.00%	60	CDS	SPN3US_0230	c.332C>T	p.T111M
	200946	Ins	-	T		91.70%	100.00%	60				
	239933	Ins	-	T		97.40%	100.00%	60				
**am43**	**Reference Position**	**Type**	**Reference Base**	**Called Base**	**Impact**	**SNP %**	**P not ref**	**Q call**	**Feature Type**	**Feature Name**	**DNA Change**	**Amino Acid Change**
	97805	SNP	G	A	Non Synonymous	99.40%	100.00%	60	CDS	SPN3US_0106	c.127G>A	p.D43N
	111698	SNP	C	T	Non Synonymous	99.60%	100.00%	60	CDS	SPN3US_0129	c.191C>T	p.S64L
	187276	SNP	C	T	Non Synonymous	100.00%	100.00%	60	CDS	SPN3US_0214	c.262G>A	p.E88K
	200945	Ins	-	T		93.00%	100.00%	60				
	215999	SNP	C	T	Nonsense	100.00%	100.00%	60	CDS	SPN3US_0241	c.2011C>T	p.Q671.
	223314	SNP	C	T		99.50%	100.00%	60				
	239933	Ins	-	T		98.30%	100.00%	60				
**am50**	**Reference Position**	**Type**	**Reference Base**	**Called Base**	**Impact**	**SNP %**	**P not ref**	**Q call**	**Feature Type**	**Feature Name**	**DNA Change**	**Amino Acid**

												**Change**
	13843	SNP	G	A		100.00%	100.00%	60				
	54485	SNP	G	A	Non Synonymous	99.50%	100.00%	60	CDS	SPN3US_0053	c.880G>A	p.G294S
	61814	SNP	C	T	Non Synonymous	99.50%	100.00%	60	CDS	SPN3US_0062	c.625C>T	p.P209S
	77967	Ins	-	T		87.90%	100.00%	60				
	94766	SNP	C	T	Non Synonymous	99.80%	100.00%	60	CDS	SPN3US_0102	c.80C>T	p.S27F
	102552	SNP	C	T	Non Synonymous	99.90%	100.00%	60	CDS	SPN3US_0115	c.367C>T	p.P123S
	135155	SNP	C	T	Non Synonymous	100.00%	100.00%	60	CDS	SPN3US_0155	c.1987C>T	p.P663S
	156070	SNP	C	T	Non Synonymous	99.80%	100.00%	60	CDS	SPN3US_0170	c.2602G>A	p.G868S
	164973	SNP	C	T	Non Synonymous	99.50%	100.00%	60	CDS	SPN3US_0178	c.490C>T	p.P164S
	170544	SNP	C	T	Nonsense	99.50%	100.00%	60	CDS	SPN3US_0186	c.1645C>T	p.Q549.
	181126	SNP	G	A	Synonymous	99.40%	100.00%	60	CDS	SPN3US_0206	c.63G>A	p.E21E
	222769	SNP	G	A	Non Synonymous	100.00%	100.00%	60	CDS	SPN3US_0248	c.116G>A	p.R39H
	239933	Ins	-	T		87.70%	100.00%	60				

Table S4 –Mass spectral counts obtained by GelCMS for the two amber mutants, *241*am11 (gp241-) and *64_112*am27 (gp64-/ gp112-), propagated on permissive (sup+) and non-permissive (sup-) strains of *Salmonella* Typhimurium (TT6675 and TT9079, respectively).

				**Total spectrum count**	
gp	Molecular weight (kDa)	am11 sup+	am11 sup-	am27 sup+	am27 sup-
8	30.3	27	22	30	30
17	15.3	21	19	21	
21	40.7	29	19	19	22
25	14.6	10	6	8	
37	14.6	6		4	
38	23.2	6	4		
41	31.8			11	
42	49.3	27		37	36
45	50.3	90	73	124	128
46	16.2	20	15	22	15
47	62.8	70	66	205	108
48	111.0	145	132	164	
49	48.1	10	14	3	
50	39.4	72	66	82	76
51	34.9	93	68	90	86
52	21.0	19	19	26	21
53	45.2	506	444	708	703

54	45.1	499	497	681	645
61	58.3	24	24	33	
62	52.0	38	39	35	
64	48.9	11	9	9	
74	12.9	45	35	45	35
75	83.9	803	1077	1723	1691
81	100.2	89	57	125	105
82	84.8	53	46	51	
83	20.0	20	20	19	18
84	32.9	76	66	66	
91	23.6	17	13	10	
94	41.6	9	11	12	17
95	17.6	17	12	11	6
97	11.9	6	6	6	3
109	17.7	12	12	28	38
123	16.5			5	
124	113.7	79	72	64	
138	29.2	42	47	40	44
139	29.8	43	46	44	36
140	31.4	29	36	31	39
141	32.6	311	319	420	326
142	30.8	55	72	85	63
143	31.9	108	107	234	134

144	30.0	76	83	102	91
145	50.8	25	24	30	31
146	36.9	32	26	34	31
147	33.7	19	17	23	17
148	53.7	45	49	47	52
149	36.2	60	69	65	54
150	33.8	31	34	35	20
151	52.2	21	25	34	18
152	36.8	70	73	80	52
153	33.8	24	33	27	16
154	50.0	29	30	22	30
155	78.3	65	61	80	61
160	18.5	124	112	280	149
167	44.8	58	42	79	
168	188.1	258	213	292	
169	149.0	244	199	252	
170	135.4	157	154	189	
171	47.6	25	27	30	
173	34.6	8	4	8	
193	19.9	12	11	9	6
202	23.5	42	40	38	
203	51.9	53	40	54	
214	28.1	61	64	77	68

218	25.2	24		23	28
223	45.3	24	25	29	
225	25.1	24	21	23	15
237	19.9	40	32	32	
238	82.1	32	29	55	
239	259.1	5	6	100	
240	59.6	14		56	38
241	159.1	110		203	152
242	10.5	8	8	18	10
243	54.6	174	164	221	217
244	27.0	10		12	13
245	30.7	46	34	28	26
246	23.9	32	39	28	39
248	21.0	18	13	14	10
255	32.7	199	188	244	
256	75.7	607	518	686	**8**
257	34.2	70	65	96	83
258	96.4	102	101	124	
259	61.0	139	136	153	
262	52.7	33	15	41	42
